# Shared Genetic Architecture Between Epigenetic Aging and Musculoskeletal Diseases

**DOI:** 10.3390/genes17080878

**Published:** 2026-07-28

**Authors:** Wei Xu, Xuanyu Zhang, Biyi Zhao, Xiaoyun Li, Ronghua Zhang

**Affiliations:** 1 College of Traditional Chinese Medicine, Jinan University, Guangzhou 510632, China; xw12137@stu2025.jnu.edu.cn (W.X.); zhaobiyi@stu.jnu.edu.cn (B.Z.); 2 Shenzhen Key Laboratory of Systems Medicine in Inflammatory Diseases, Zhongshan School of Medicine, Sun Yat-sen University, Shenzhen Campus, Shenzhen 518107, China; zhangxy955@mail2.sysu.edu.cn; 3 Department of Medical Informatics and Neurobiology Research Center, Zhongshan School of Medicine, Sun Yat-sen University, Shenzhen 518107, China; 4 College of Pharmacy, Jinan University, Guangzhou 510632, China; 5 Guangdong Provincial Key Laboratory of Traditional Chinese Medicine Informatization, Guangzhou 510632, China; 6 State Key Laboratory of Bioactive Molecules and Druggability Assessment, Guangdong Basic Research Center of Excellence for Natural Bioactive Molecules and Discovery of Innovative Drugs, The First Affiliated Hospital, Jinan University, Guangzhou 510632, China

**Keywords:** epigenetic age acceleration, musculoskeletal diseases, DNA methylation clocks, LD score regression, cross-trait pleiotropic locus mapping, colocalization, multivariable Mendelian randomization

## Abstract

Background: The directional relationship between epigenetic age acceleration (EAA) and musculoskeletal disease remains unresolved. This study integrated bidirectional Mendelian randomization (MR) with multi-layer genomic evidence to evaluate directionality, shared genetic architecture, and robustness to instrument definition. Methods: Four EAA clocks (IEAA, PhenoAA, HannumAA, and GrimAA) and ten musculoskeletal phenotypes were analyzed in a 10 × 4 bidirectional two-sample MR design. EAA instruments underwent GRCh37 functional annotation, genome-wide-significant external-association screening for the index variants and European linkage-disequilibrium proxies, pair-specific Steiger filtering, and conservative Set A/B/C sensitivity analyses. The juvenile-arthritis reverse models underwent instrument-flow reconstruction, strength assessment, liability-scale directionality testing, and minimum-detectable-effect analysis. Additional analyses comprised LD score regression (LDSC), PLACO+ cross-trait locus mapping, Bayesian colocalization, multivariable MR (MVMR) with exact-SNP matched univariable comparators, and integrated evidence synthesis. Results: Forward MR yielded two nominal HannumAA associations. The inverse HannumAA–spondyloarthritis estimate remained directionally consistent across the original, Steiger-filtered, and conservative external-association-filtered sets, whereas the HannumAA–pain-in-thoracic-spine estimate lost nominal significance in the conservative set; no forward result survived correction across 40 tests. GrimAA forward estimates were sensitive to use of the fallback instrument threshold. Reverse MR identified ten nominal associations. For juvenile arthritis, three harmonized instruments had *F* statistics of 51.25–102.35; liability-scale Steiger comparisons supported the tested direction under all 16 outcome-by-prevalence combinations, although the 788-case discovery GWAS and possible winner’s curse remained important limitations. LDSC identified FDR-significant positive genetic correlations of GrimAA with hip osteoarthritis (*r_g* = 0.267, *p* = 8.49 × 10^−5^, *q* = 0.0019) and knee osteoarthritis (*r_g* = 0.269, *p* = 9.52 × 10^−5^, *q* = 0.0019). PLACO+ identified 738 genome-wide-significant cross-trait variants and 65 independent loci; six of 37 evaluable loci showed strong colocalization. Of 96 MVMR models, 43 had primary-exposure conditional *F* ≥ 10, and 32 also had candidate-trait conditional *F* ≥ 10. After exact-SNP matching, the 43 primary-strength models were operationally classified as 35 partially attenuated and eight independent-signal models, with no fully attenuated model; no adjusted association survived multiplicity correction. Conclusions: The results support a prioritized genomic map with substantial instrument- and model-specific uncertainty. Disease-to-clock signals were richer than clock-to-disease signals, GrimAA shared polygenic architecture with osteoarthritis, and selected loci showed strong shared-variant evidence, while the MR and MVMR findings remained unsuitable for definitive causal or mediation claims.

## 1. Introduction

Musculoskeletal diseases are major contributors to disability, pain, frailty, and loss of independence across the life course [1,2,3,4]. Their biology is heterogeneous: fractures reflect bone strength, falls, trauma susceptibility, endocrine status, and tissue repair; osteoarthritis and arthrosis involve cartilage, subchondral bone, synovium, biomechanics, and low-grade inflammation; inflammatory arthropathies involve immune dysregulation; and chronic axial pain may integrate structural, inflammatory, neurological, and psychosocial components [5,6,7,8,9,10,11,12]. This heterogeneity makes musculoskeletal disease an informative setting in which to examine whether molecular aging readouts capture upstream susceptibility, downstream disease burden, or shared systemic biology [13,14,15].

Epigenetic age acceleration (EAA) measures provide methylation-derived indices of biological aging, but different clocks emphasize different domains [13,14,16]. IEAA was designed to capture a more cell-intrinsic component of methylation aging; HannumAA is a blood-derived aging measure, PhenoAA is anchored to clinical biomarker-defined phenotypic aging, and GrimAA is enriched for methylation surrogates related to mortality and healthspan [16,17,18,19]. These differences are important for musculoskeletal research because immune activation, metabolic dysfunction, chronic pain, reduced mobility, body composition, and tissue degeneration may influence each clock differently [15,20,21,22]. All four EAA GWAS used in this study were derived from peripheral-blood DNA methylation [23]. These clocks therefore capture systemic and hematopoietic aging signals and should not be assumed to measure aging directly within skeletal muscle, bone, cartilage, or synovium.

The central inferential question is directional. If EAA contributes to musculoskeletal disease liability, accelerated methylation aging may mark pathways that precede structural degeneration, inflammatory disease, fracture risk, or pain susceptibility [13,24,25]. Conversely, if musculoskeletal disease liability contributes to altered EAA, methylation-clock differences may partly reflect the systemic consequences of disease, including inflammation, immune-cell remodeling, treatment exposure, physical inactivity, pain-related stress, or metabolic change [20,21,26,27]. Bidirectional two-sample MR is useful for this question because germline genetic instruments are fixed before disease onset and are less vulnerable to conventional reverse causation than observational methylation–disease associations [28,29,30].

A single MR association, however, does not fully resolve the genetic architecture linking aging clocks and musculoskeletal phenotypes [31,32,33]. Directional MR estimates may coexist with genome-wide polygenic sharing, locus-level pleiotropy, linkage disequilibrium between distinct causal variants, or attenuation after adjustment for plausible mechanism traits [34,35,36,37,38]. Triangulating across LDSC, PLACO+, colocalization, and MVMR can therefore separate several biologically different scenarios: diffuse shared genetic background, candidate cross-trait loci, shared regional causal-variant models, and associations that are sensitive to inflammatory, metabolic, functional, or bone-related covariates [34,35,36,37,39,40].

In this study, we evaluated four EAA clocks and 10 musculoskeletal phenotypes using a bidirectional MR framework complemented by genetic correlation, pleiotropic locus mapping, Bayesian colocalization, and mechanism-oriented MVMR. The objective was to identify which EAA–musculoskeletal relationships are most consistent with robust genomic prioritization, which appear directionally asymmetric, and which remain hypothesis-generating because of heterogeneity, incomplete regional variant coverage, or limited conditional instrument strength.

## 2. Methods

### 2.1. Study Design

**Figure 1:**
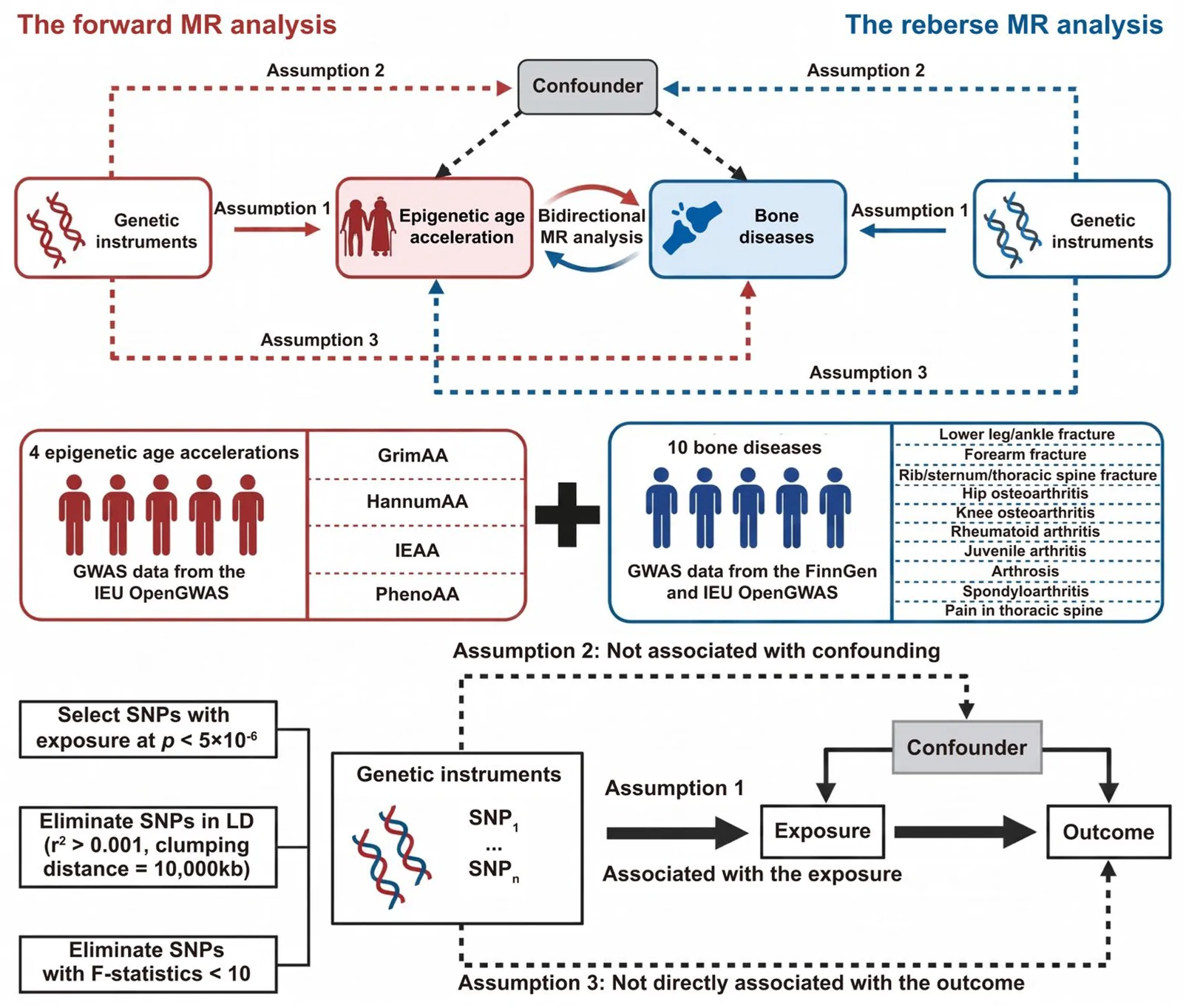
Overview of the bidirectional MR study design. The schematic illustrates forward and reverse-MR analyses using genetic instruments derived from GWAS datasets for EAA and 10 musculoskeletal phenotypes. Assumptions 1–3 represent the core MR assumptions: genetic association with the exposure, independence from confounders, and influence on the outcome only through the exposure.

A bidirectional two-sample MR design was used. In the forward direction, each EAA trait served as the exposure, and each musculoskeletal phenotype served as the outcome. In the reverse direction, each musculoskeletal phenotype served as the exposure and each EAA trait served as the outcome. With 10 musculoskeletal phenotypes and four EAA clocks, the analytical matrix comprised 40 forward tests and 40 reverse tests, for 80 directional tests overall. The overall study design is shown in Figure 1.

The analytical framework also included genome-wide genetic correlation by LDSC, cross-trait pleiotropic variant mapping by PLACO+, locus-level colocalization by coloc.abf, and mechanism-oriented MVMR. The integrated evidence table combined MR, LDSC, PLACO+, colocalization, and MVMR features for all 80 directional rows.

### 2.2. Data Sources and Trait Definitions

All summary statistics were obtained from publicly accessible GWAS resources [41,42]. Musculoskeletal phenotypes were drawn from FinnGen and IEU OpenGWAS records and were restricted to European-ancestry cohorts. The disease panel covered three fracture phenotypes, osteoarthritis/arthrosis phenotypes, inflammatory arthropathies, juvenile arthritis, and pain in the thoracic spine. Definitions and measurement characteristics of the four EAA clocks are summarized in Table 1, and the GWAS identifiers, sample sizes, and PubMed source records for the musculoskeletal and EAA traits are summarized in Table 2 [23,42,43].

Across the disease GWAS datasets, total sample size ranged from 166,884 for pain in the thoracic spine to 417,596 for the hip osteoarthritis GWAS, and case counts ranged from 788 for juvenile arthritis to 39,427 for hip osteoarthritis. The four EAA GWAS datasets also came from European-ancestry samples, with sample sizes ranging from 34,449 to 34,467. Juvenile arthritis had the smallest case count (*n* = 788). Although retained variants were required to satisfy the prespecified per-variant *F*-statistic threshold after harmonization, the small discovery sample may reduce SNP-exposure precision, limit statistical power, and increase susceptibility to winner’s curse in reverse MR.

### 2.3. Instrument Selection, Clumping, and Weak-Instrument Filtering

Instrument selection followed a uniform pre-specified procedure for each exposure trait [29,44]. Candidate SNPs were first selected at *p* < 5 × 10^−8^ [29,32]. If no analyzable instruments were available at this threshold, a fallback threshold of *p* < 5 × 10^−6^ was applied. Linkage-disequilibrium pruning used *r*^2^ < 0.001 within a 10,000 kb window [41,45,46]. After harmonization, per-variant *F* statistics were used to exclude weak instruments (*F* ≥ 10 retained) [32].

Exposure and outcome summary statistics were harmonized using allele-alignment rules, including effect-allele matching and strand-consistency checks [29,41]. Palindromic or otherwise incompatible variants were excluded when strand orientation could not be resolved.

The EAA instruments were selected because they predicted blood-derived clock residuals at the GWAS level; they were not restricted to variants with an established direct effect on clock CpGs or a single methylation-aging pathway. Accordingly, the instruments represent genetic liability to the composite EAA traits. Horizontal effects through immune regulation, hematopoietic cell composition, metabolic pathways, smoking-related components, or other biological processes cannot be excluded by instrument selection alone.

To assess the biological interpretability of the EAA instruments and the possibility of horizontal pleiotropy, all 61 clock instruments were positionally annotated on GRCh37 [47]. Genome-wide-significant external associations (*p* < 5 × 10^−8^) were screened for the index variants and European linkage-disequilibrium proxies (*r*^2^ ≥ 0.80 within ±1 Mb) [48]. External phenotypes were assigned to predefined, non-mutually exclusive biological domains using ontology terms, complete phenotype definitions, and exact-phrase rules. Associations related to the clock definition and associations supporting a potential outcome pathway outside the EAA construct were evaluated independently. The original instrument set was designated Set A; Set B retained pair-specific Steiger-consistent variants [49]; and Set C further excluded variants with high-confidence direct or proxy evidence for a plausible independent pathway to the musculoskeletal outcome. Set C was treated as a conservative sensitivity analysis rather than a replacement primary analysis. For each set, the 40 forward IVW tests were evaluated using Bonferroni correction and the Benjamini–Hochberg false-discovery rate [50]. Because 17 of 21 GrimAA instruments were selected at the fallback threshold, an additional GrimAA sensitivity analysis was restricted to the four instruments meeting *p* < 5 × 10^−8^.

### 2.4. MR Estimation and Sensitivity Analyses

Primary causal estimation used inverse-variance weighting when more than one valid SNP remained [29,30,51]. When only a single SNP was available, the Wald ratio was used. Forward-direction effects are reported as odds ratios with 95% confidence intervals after exponentiation of log-odds estimates. Reverse-direction effects are reported as beta coefficients with 95% confidence intervals on the native EAA outcome scale.

When SNP support was adequate, complementary estimators included MR-Egger, weighted median, simple mode, and weighted mode [33,52,53]. Heterogeneity was assessed using Cochran-type *Q* statistics from IVW and MR-Egger models, directional horizontal pleiotropy was evaluated using the MR-Egger intercept, and leave-one-out analysis was used to assess single-variant influence. For associations selected for extended sensitivity visualization, each plotted panel paired SNP-level scatter estimates with leave-one-out estimates. The scatter component was used to compare estimator direction and slope across IVW, MR-Egger, weighted median, simple mode, and weighted mode where available. The leave-one-out component was used to identify whether the overall estimate was dependent on removal of one influential SNP. These plots were interpreted jointly with the numeric heterogeneity and Egger-intercept diagnostics in Table 3; visual direction alone was not treated as sufficient evidence of robustness.

#### Juvenile Arthritis Instrument Strength and Detectability

Because the juvenile-arthritis GWAS contained only 788 cases, the reverse-MR instrument flow was reconstructed from selection through harmonization. Three variants (rs115681000, rs116080026, and rs9266804) constituted the exact primary set; rs2073722 was excluded by harmonise_data(action = 2) because of incompatible alleles. Per-variant strength was calculated as *F* = (*β_exposure_*/*SE_exposure_*)^2^, and the effective case–control sample size was calculated as 4/(1/*N_case_* + 1/*N_control_*). Juvenile-arthritis variance explained was converted to the liability scale [54] under a primary standardized European pediatric prevalence of 0.0702%, with 0.056%, 0.0837%, and the FinnGen period prevalence of 0.44% examined as sensitivity assumptions [42,55,56,57]. Steiger directionality [49] compared the summed liability-scale exposure *R*^2^ with the summed continuous-outcome *R*^2^ for the same three-SNP set. Minimum detectable effects were calculated for 80% and 90% power at *α* = 0.05 and *α* = 0.0125; prospective power curves used an absolute effect grid of 0.05–0.60 SD. Winner’s curse [58] was considered separately from conventional weak-instrument bias by comparing the archived coefficients with the later overlapping FinnGen release and by auditing the availability of independent replication and selection-adjusted analyses.

### 2.5. LDSC Genetic Correlation

To quantify genome-wide shared genetic architecture, LDSC was applied to all 40 undirected EAA–musculoskeletal trait pairs [34,35]. Summary statistics were harmonized to HapMap3 variants and analyzed using pre-computed European LD scores derived from the 1000 Genomes Project European reference population [34,45]. Observed-scale SNP heritability, genetic correlation coefficients (*r*_g_), standard errors, Z statistics, and two-sided *p* values were estimated. False-discovery-rate control used Benjamini–Hochberg *Q* values, with *Q* < 0.05 considered FDR significant.

### 2.6. PLACO+ Cross-Trait Pleiotropic Variant Mapping

PLACO+ was used to identify variants showing cross-trait association with EAA and musculoskeletal phenotypes under a composite null framework [36]. The method identifies variants associated with both traits but does not, by itself, establish a shared causal variant or causal direction. Prior to testing, summary statistics were harmonized; ambiguous variants and palindromic SNPs with intermediate effect-allele frequencies were excluded. The main MHC interval on chromosome 6 (chr6:28,477,797–33,448,354, GRCh37/hg19) was removed to reduce spurious signals from complex LD.

PLACO+ was applied to 19 prioritized EAA–musculoskeletal pairs. Genome-wide-significant PLACO+ variants were defined at *p* PLACO+ < 5 × 10^−8,^ and independent pleiotropic loci were obtained by PLINK clumping using the 1000 Genomes European reference panel at *r*^2^ < 0.001 within 10,000 kb [45,46].

### 2.7. Bayesian Colocalization

To evaluate whether PLACO+ loci were compatible with shared causal-variant models rather than nearby but distinct association signals, Bayesian colocalization was performed using coloc.abf from the R package coloc (version 5.2.3) [37]. For each of the 65 independent PLACO+ loci, a ±250 kb regional window centered on the lead SNP was extracted from both the corresponding EAA and musculoskeletal standardized summary statistics.

Alleles were aligned to the EAA strand, flipped variants had musculoskeletal effect sizes negated, ambiguous palindromic variants were excluded, and loci with fewer than five harmonized SNPs were treated as not evaluable. Posterior probabilities were computed for *H*_0_–*H*_4_, where *H*_4_ represents a shared causal variant. Evidence was classified as strong when PP.H_4_ ≥ 0.75, moderate when PP.*H*_4_ was 0.30–0.69, and no evidence when PP.H_4_ < 0.30. Rows encoded with PP.H_0_ = 1.0 and PP.H_4_ = 0.0 because zero harmonized SNPs remained were treated as non-evaluable coverage failures, not as biological null results.

The six strongly colocalized lead variants reported in Table 4 were positionally annotated on GRCh37 using Ensembl Variant Effect Predictor output [47] and Ensembl GRCh37 gene models. All transcript consequences were retained and linked to their corresponding genes. When a variant overlapped a noncoding gene, the nearest protein-coding gene was additionally reported by distance to the gene body. These annotations provide positional locus context and were not used to infer causal target genes.

### 2.8. Multivariable MR

MVMR was performed for MR-prioritized EAA–musculoskeletal pairs by adding one candidate trait at a time: BMI, CRP, IL-6, TNF-R1, white blood cell count, HbA1c, grip strength, or heel bone mineral density [38,39,40]. Model-specific allele-valid SNP matrices were analyzed using ivw_mvmr with the exposure–covariance term set to zero because cohort-level overlap and cross-trait covariance estimates were unavailable. Conditional *F* statistics for both the primary and candidate exposures and residual *Q* statistics were obtained using strength_mvmr and pleiotropy_mvmr, respectively. Models with primary-exposure conditional *F* ≥ 10 formed the primary-strength-restricted subset; models in which both conditional *F* statistics were ≥10 formed a dual-strength sensitivity subset.

For every MVMR model, the univariable comparator was re-estimated using exactly the same allele-valid SNP rows, primary-exposure associations, outcome associations, and inverse-outcome-variance weights as the adjusted model. Attenuation was calculated as 100 × [1 − |*β*_MVMR|/|*β*_matched|]. The established operational categories were retained: an independent signal required concordant directions, MVMR *p* < 0.10, and attenuation < 40%; a partially attenuated result required concordant directions and attenuation < 80% unless the independent-signal rule was met; all remaining results were classified as fully attenuated. Negative attenuation, sign reversal, candidate-exposure weakness, residual heterogeneity, and large original-to-matched effect shifts were recorded separately. The categories describe numerical changes in effect magnitude and do not establish mediation, mechanism-specific causality, or biological independence. MVMR association and *Q*-test *p* values were adjusted by Bonferroni correction and the Benjamini–Hochberg procedure within the complete, primary-strength, and dual-strength model sets.

### 2.9. Integrated Evidence Synthesis and Visualization

The integrated evidence matrix combined the primary MR result, LDSC genetic correlation, PLACO+ locus count, best coloc PP.H_4_, and MVMR attenuation pattern for all 80 directional rows. The integrated evidence map uses column-wise min–max scaling for display only because the evidence components are on different scales, including −log_10_(MR *p*), −log_10_(LDSC *Q*), log1p(PLACO loci), best coloc PP.H_4_, and an ordinal MVMR score. All 10 musculoskeletal phenotypes were retained as disease nodes regardless of whether a phenotype entered a PLACO+ prioritized pair. Colored or dashed edges were drawn only for the 19 PLACO+ prioritized EAA–disease pairs; therefore, absence of a colored edge indicates that the pair was not part of the PLACO+/colocalization layer or lacked evaluable colocalization evidence, not that the phenotype was absent from the study.

### 2.10. Statistical Environment and Reproducibility

Primary MR analyses were conducted in R 4.1.0 using standardized analytical procedures for instrument extraction, harmonization, MR estimation, diagnostic testing, and figure generation [41,44]. Multi-layer genomic orchestration and visualization used Python 3.13.2. Exact-SNP-matched MVMR estimation used R 4.3.3 with MVMR 0.4.6, while Bayesian colocalization was implemented using coloc.abf in R.

## 3. Results

### 3.1. Dataset Coverage and Primary MR Scope

The analysis completed the full 10 × 4 bidirectional MR matrix in both directions. This phenotype set included fracture phenotypes, osteoarthritis phenotypes, arthrosis, inflammatory arthropathies, juvenile arthritis, and pain in the thoracic spine. The resulting 80 directional tests provided the base map for downstream sensitivity and multi-layer genomic triangulation.

### 3.2. Forward Direction: EAA to Musculoskeletal Outcomes

**Figure 2:**
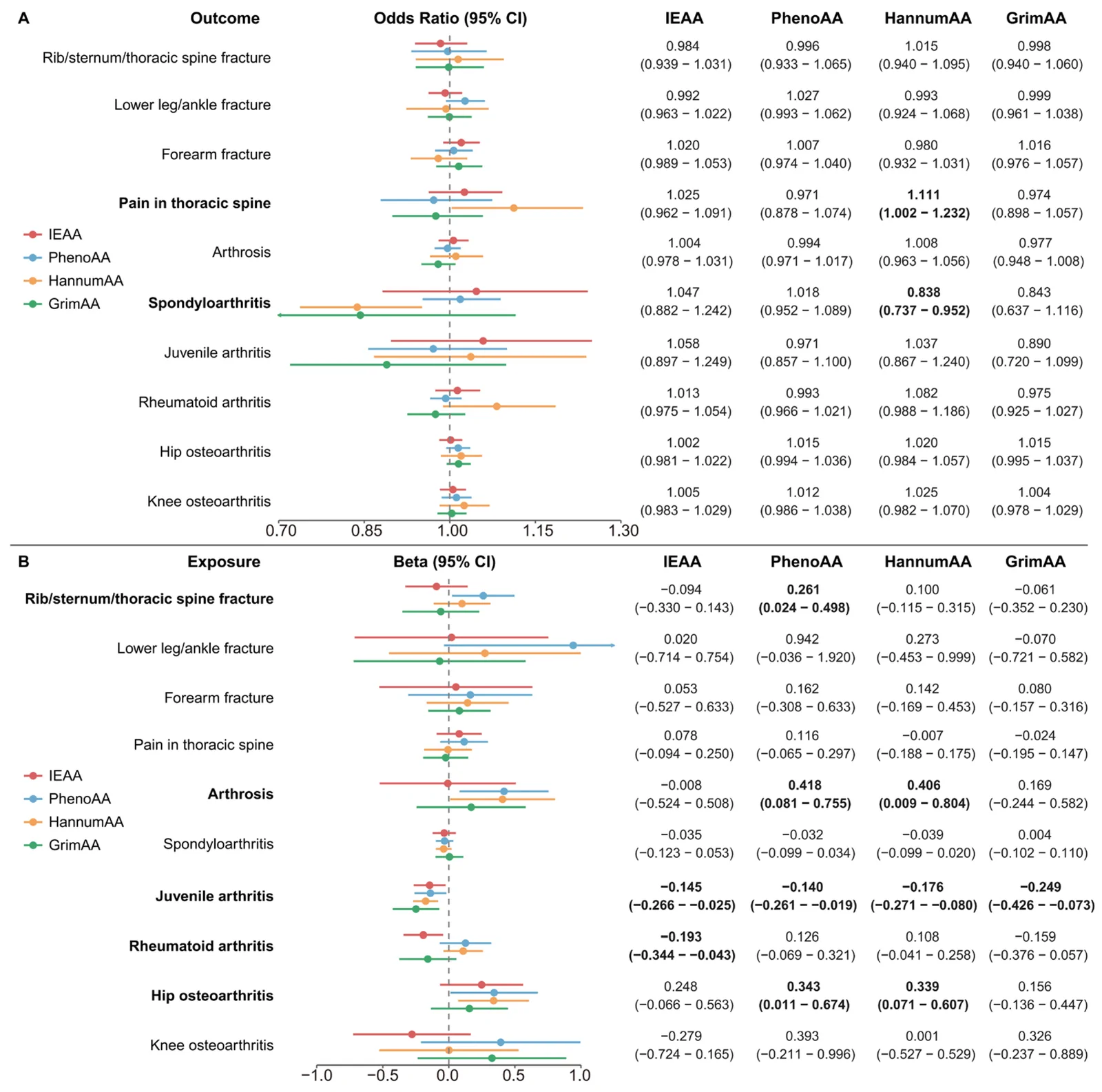
Bidirectional primary MR effect panels. Panel (**A**) shows forward-direction effects with EAA clocks as exposures and musculoskeletal outcomes as outcomes. Panel (**B**) shows reverse-direction effects with musculoskeletal phenotypes as exposures and EAA clocks as outcomes.

In the forward direction, the primary MR effect panel showed that most estimates clustered close to the null, indicating that genetically predicted EAA did not show a generalized pattern of strong causal influence on musculoskeletal disease liability (Figure 2A). The two nominal forward associations both involved HannumAA. Higher genetically predicted HannumAA was associated with higher pain in thoracic spine liability (OR 1.111, 95% CI 1.002 to 1.232), and its sensitivity profile showed directionally coherent estimator lines with no single leave-one-out SNP driving the association (Supplementary Figure S1A). Higher genetically predicted HannumAA was also associated with lower spondyloarthritis liability (OR 0.838, 95% CI 0.737 to 0.952), but this association was accompanied by significant heterogeneity in the diagnostic table and should be interpreted as a heterogeneous forward signal rather than a stable protective effect (Supplementary Figure S1B; Table 3).

Fracture-related forward estimates were uniformly modest across clocks. Degenerative and inflammatory phenotypes were also largely null in the forward direction apart from the two HannumAA signals.

#### Instrument Annotation and Forward-MR Robustness

The annotation screen retained 709 genome-wide-significant external-association records for the 61 EAA instruments or their qualifying European proxies. Twenty-five instruments met the prespecified high-confidence criterion for exclusion from Set C. The resulting Set A, Set B, and Set C analyses were available for all 40 forward trait pairs. The instrument-level annotation and external-association evidence are summarized in Supplementary Figure S4.

For HannumAA to spondyloarthritis, the estimate remained inverse in Set A (OR = 0.838, 95% CI 0.737–0.952, *p* = 0.0065), Set B (OR = 0.873, 95% CI 0.796–0.958, *p* = 0.0042), and Set C (OR = 0.871, 95% CI 0.769–0.986, *p* = 0.0295). Removal of the Steiger-inconsistent variant reduced the IVW heterogeneity from *p* = 0.0418 in Set A to *p* = 0.944 in Set B. For HannumAA to pain in thoracic spine, the positive estimate was unchanged in Sets A and B (OR = 1.111, 95% CI 1.002–1.232, *p* = 0.0460) but was less precise and no longer nominally significant in Set C (OR = 1.134, 95% CI 0.974–1.321, *p* = 0.106). GrimAA to hip osteoarthritis reached nominal significance only in Set C (OR = 1.027, *p* = 0.0320), indicating filtering sensitivity. No forward association survived Bonferroni or false-discovery-rate correction within any instrument set. In the GrimAA strict-threshold comparison, 17 of 30 set-specific contrasts changed direction, and two changed nominal-significance status, demonstrating substantial dependence on the fallback-inclusive instrument definition. Set A/B/C robustness and GrimAA instrument-threshold sensitivity are shown in Supplementary Figures S5 and S6, respectively.

### 3.3. Reverse Direction: Musculoskeletal Traits to EAA

The reverse primary effect panel showed a denser pattern of nominal associations than the forward direction (Figure 2B). Juvenile-arthritis liability was associated with lower IEAA, PhenoAA, HannumAA, and GrimAA; the four juvenile-arthritis sensitivity panels show the same inverse direction across clocks but different instrument sets and leave-one-out profiles (Supplementary Figure S2A–D). Rheumatoid arthritis liability was associated with lower IEAA (Supplementary Figure S3C). Arthrosis liability was associated with higher PhenoAA and HannumAA (Supplementary Figure S3A,B), and hip osteoarthritis liability was associated with higher PhenoAA and HannumAA (Supplementary Figure S3D,E). Rib/sternum/thoracic spine fracture liability was associated with higher PhenoAA in the MR table, but the corresponding reverse-direction sensitivity panel could not be generated because no harmonized SNPs were available for the rib/sternum/thoracic spine fracture to PhenoAA plotting set; therefore, this result is interpreted from the MR table and numeric diagnostics rather than from a plotted sensitivity panel. The juvenile-arthritis estimates should be treated as exploratory because the exposure GWAS contained only 788 cases and the reverse models relied on sparse-instrument sets.

These reverse associations were not distributed evenly across clocks. Positive degenerative signals concentrated in PhenoAA and HannumAA, whereas juvenile arthritis showed inverse associations across all four clocks. This clock specificity supports interpretation by phenotype–clock pair rather than by a single generic EAA construct.

### 3.4. Sensitivity Diagnostics for Nominal MR Associations

The heterogeneity and pleiotropy diagnostics for associations taken into extended sensitivity analysis are summarized in Table 3. Extended sensitivity analyses supported pair-specific interpretation rather than a single global EAA–musculoskeletal model. For the forward analyses, HannumAA to pain in the thoracic spine had a modest positive IVW estimate and a visually consistent scatter/leave-one-out profile (Supplementary Figure S1A), whereas HannumAA to spondyloarthritis had an inverse IVW estimate but significant heterogeneity, making this panel a hypothesis-generating forward result rather than a definitive protective association (Supplementary Figure S1B; Table 3).

#### Juvenile Arthritis Instrument Strength and Detectability

Instrument-flow reconstruction showed that the four archived candidate variants were reduced to a three-SNP primary set because rs2073722 had incompatible alleles. The three retained variants had *F* statistics of 51.25–102.35 (median 88.96), while the effective case–control sample size was 3137.7 despite the nominal total sample size of 173,622. The three-SNP IVW estimates were −0.145 for IEAA (*p* = 0.0183), −0.140 for PhenoAA (*p* = 0.0235), −0.176 for HannumAA (*p* = 3.04 × 10^−4^), and −0.249 for GrimAA (*p* = 0.00569).

Across the four prevalence assumptions, total juvenile-arthritis liability *R*^2^ ranged from 0.0563 to 0.0565, and the exposure-to-outcome *R*^2^ ratio exceeded one in all 16 outcome-by-prevalence comparisons. At the standardized European pediatric prevalence, the 80% minimum-detectable-effect was approximately 0.062 SD at *α* = 0.05 and 0.074 SD at *α* = 0.0125; the corresponding 90% values were approximately 0.072 and 0.083 SD. Within the four juvenile-arthritis-to-EAA comparisons, all four estimates met the Benjamini–Hochberg threshold and the HannumAA and GrimAA estimates met the four-comparison Bonferroni threshold; these secondary within-phenotype corrections do not replace multiplicity considerations across the complete bidirectional MR matrix. The later overlapping FinnGen release showed directionally concordant but smaller SNP-exposure coefficients, so winner’s curse could not be excluded. Instrument flow, liability-scale directionality, and prospective detectability are summarized in Supplementary Figure S7.

For juvenile arthritis, the three-SNP models showed no IVW heterogeneity for IEAA (*Q p* = 0.608), PhenoAA (*Q p* = 0.469), HannumAA (*Q p* = 0.703), or GrimAA (*Q p* = 0.122). MR-Egger residual heterogeneity was absent for IEAA, PhenoAA, and HannumAA and was nominal for GrimAA (*Q p* = 0.041), but each MR-Egger model had only one residual degree of freedom. Egger-intercept *p* values ranged from 0.512 to 0.989, providing no evidence of directional pleiotropy within the limited three-instrument setting.

For the additional reverse sensitivity profiles, arthrosis to PhenoAA and arthrosis to HannumAA were both positive but differed in heterogeneity, with arthrosis to HannumAA showing stronger heterogeneity (Table 3; Supplementary Figure S3A,B). Rheumatoid arthritis to IEAA was inverse and should be interpreted separately from the osteoarthritis/arthrosis panels because its exposure is inflammatory arthritis rather than a degenerative joint phenotype (Supplementary Figure S3C). Hip osteoarthritis to HannumAA and hip osteoarthritis to PhenoAA were both positive and directionally stable in the plotted sensitivity set, supporting their retention as reverse-direction secondary signals while still requiring multiple-testing caution (Supplementary Figure S3D,E).

### 3.5. Genome-Wide Genetic Correlation by LDSC

LDSC evaluated 40 EAA–musculoskeletal genetic correlations. Two correlations survived FDR correction: GrimAA with hip osteoarthritis (*r*_g_ = 0.267, SE = 0.0679, *p* = 8.49 × 10^−5^, *Q* = 0.0019) and GrimAA with knee osteoarthritis (*r*_g_ = 0.269, SE = 0.0689, *p* = 9.5249 × 10^−5^, *Q* = 0.0019). There were nine nominal correlations at *p* < 0.05. These results place the strongest genome-wide shared architecture in the GrimAA–osteoarthritis axis rather than in the nominal MR findings alone (Supplementary Figure S8).

Nominal correlations included PhenoAA with rheumatoid arthritis, PhenoAA with arthrosis, PhenoAA with hip osteoarthritis, GrimAA with arthrosis, GrimAA with rib/sternum/thoracic spine fracture, HannumAA with rib/sternum/thoracic spine fracture, and IEAA with forearm fracture. Most other pairwise correlations were not significant, supporting a selective rather than global pattern of shared genetic architecture (Supplementary Table S4).

### 3.6. PLACO+ Cross-Trait Pleiotropic Loci

**Figure 3:**
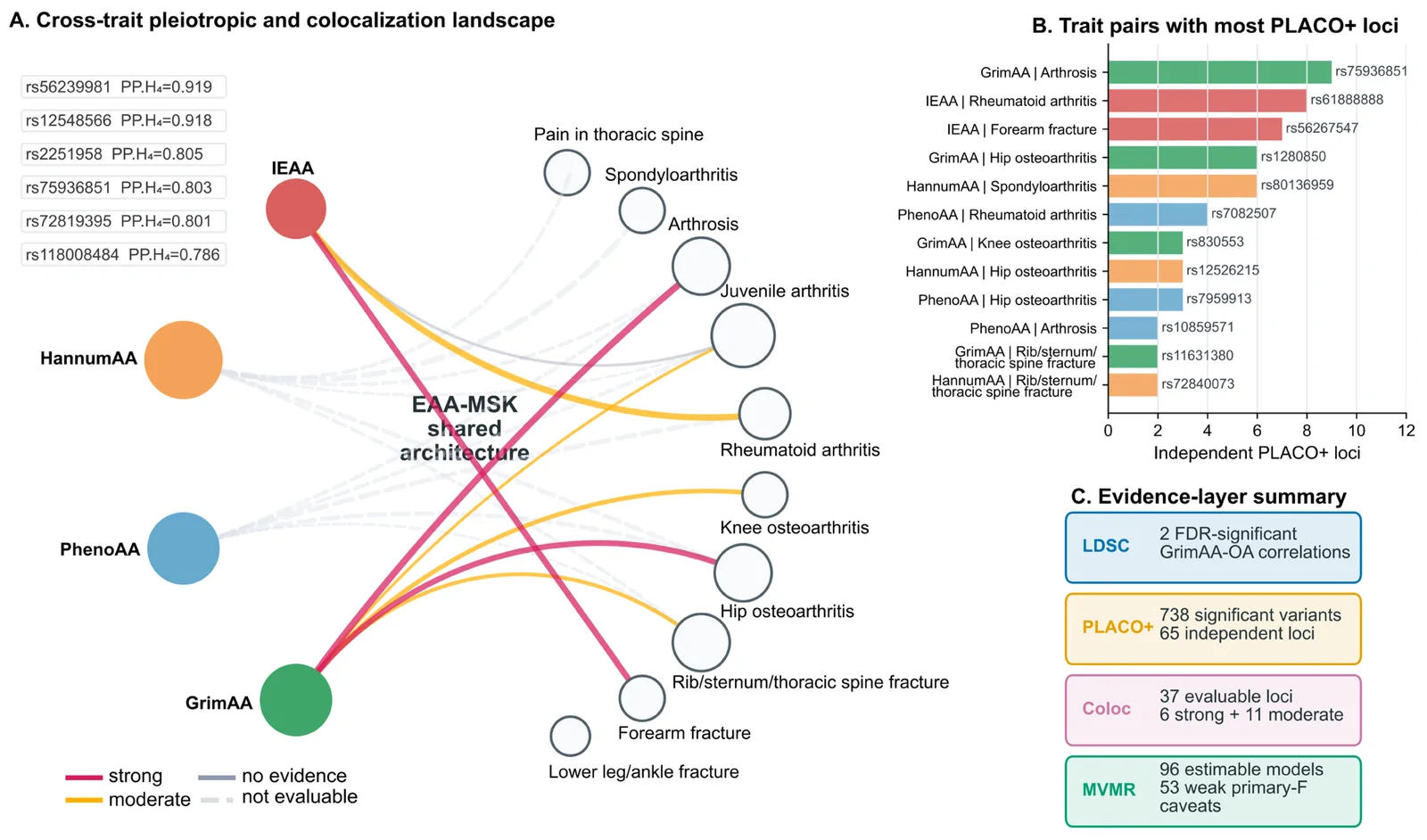
Shared genetic architecture and pleiotropic locus landscape. Panel (**A**) shows all 10 musculoskeletal phenotypes from Table 2 as disease nodes and overlays the subset of EAA–disease connections evaluated by PLACO+ and colocalization; disease nodes without PLACO+ prioritized pairs remain visible without colored connections. Line color denotes coloc evidence class, line width reflects independent PLACO+ locus burden, and dashed pale connections denote loci that were not evaluable by colocalization. Panel (**B**) ranks trait pairs by independent PLACO+ locus count and labels the top PLACO+ SNP for each pair. Panel (**C**) summarizes the four genomic evidence layers: LDSC, PLACO+, colocalization, and MVMR.

PLACO+ was applied to 19 prioritized EAA–musculoskeletal pairs and identified 738 genome-wide-significant cross-trait variants. Clumping yielded 65 independent pleiotropic loci. These loci provide candidate cross-trait association signals, but PLACO+ does not distinguish shared causal variants from linkage or nearby independent signals. The cross-trait locus burden and top pair structure are shown in Figure 3A,B and Supplementary Figure S9, with complete pair and locus records in Supplementary Tables S5 and S6.

Several biologically interpretable candidate regions require careful wording. The *GDF5* promoter/5′ untranslated-region variant rs143383 was a PLACO+ signal for HannumAA with arthrosis (*p*_PLACO_+ = 2.69 × 10^−10^) and HannumAA with hip osteoarthritis (*p*_PLACO+_ = 1.77 × 10^−9^). In the standardized files, the effect allele was a, and the other allele was g; no strand conversion to the literature allele labels was inferred. Colocalization for the rs143383 regions was not evaluable because zero harmonized regional SNPs remained, so this should be described as a PLACO+ candidate signal rather than a shared causal variant.

The MHC-flanking rs80136959 signal on chromosome 6 was the strongest PLACO+ finding for HannumAA with spondyloarthritis (*p*_PLACO+_ = 3.21 × 10^−30^) and was also observed for HannumAA with juvenile arthritis (*p*_PLACO+_ = 4.70 × 10^−10^). Because this locus is close to the excluded MHC interval and HannumAA-linked coloc regions were not evaluable, it should be framed as an immune-adjacent candidate association, not proof of an independent shared causal variant. *ASCL1*-adjacent PhenoAA signals rs10859571 and rs7959913 for arthrosis and hip osteoarthritis were similarly PLACO+ candidates without evaluable colocalization support.

### 3.7. Colocalization of PLACO+ Loci

Colocalization was attempted for all 65 independent PLACO+ loci. Of these, 37 were evaluable after regional extraction and allele harmonization, and 28 were not evaluable because zero harmonized SNPs remained. Among the evaluable loci, six had strong colocalization evidence, 11 had moderate evidence, and 20 evaluable loci had no evidence. Non-evaluable rows were treated as coverage failures rather than biological nulls (Supplementary Figure S10; Supplementary Table S7).

**Figure 4:**
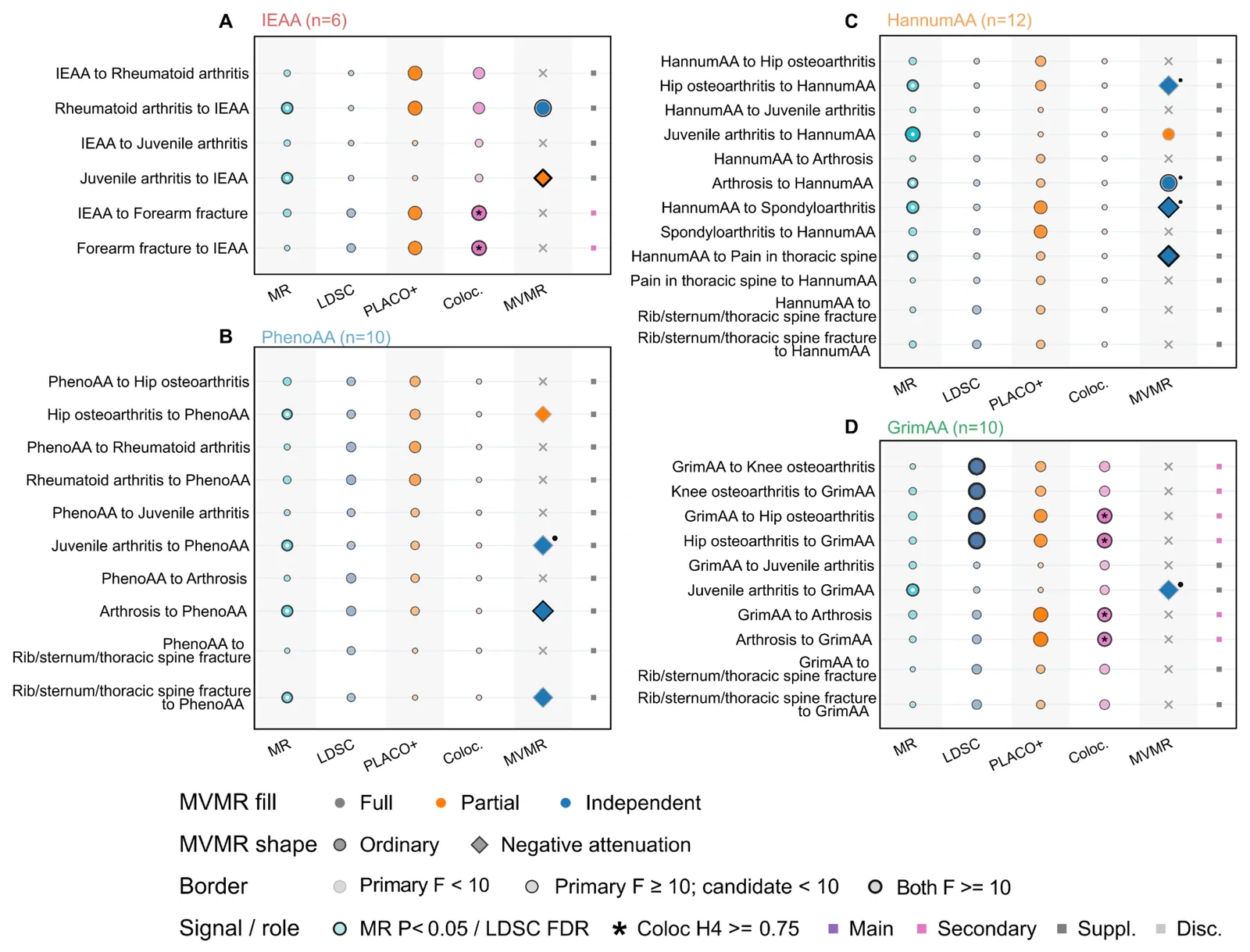
Integrated evidence maps for prioritized directional EAA–musculoskeletal rows. Panels (**A**–**D**) show IEAA, PhenoAA, HannumAA, and GrimAA, respectively. Columns represent MR, LDSC, PLACO+, colocalization, and MVMR. Within the first four columns, point area and color intensity encode the normalized evidence score. An MR ring denotes nominal MR evidence, an LDSC ring denotes FDR-significant genetic correlation, and an asterisk denotes strong colocalization (PP.*H*_4_ ≥ 0.75). In the MVMR column, gray, orange, and blue indicate fully attenuated, partially attenuated, and independent signal, respectively; diamonds denote negative attenuation and triangles denote sign reversal. MVMR outlines encode conditional-strength strata. The square at the right identifies the manuscript role. Normalization is column-specific and intended for within-column visualization only. An additional outer ring denotes residual *Q*-test *p* < 0.05, a small black dot denotes raw MVMR *p* < 0.05, and × denotes that no MVMR model was tested for that directional row.

The six strong colocalized loci were concentrated in three trait-pair categories: GrimAA with hip osteoarthritis, GrimAA with arthrosis, and IEAA with forearm fracture. These loci support shared causal-variant models at the regional level but do not establish causal direction. They also do not rescue non-evaluable HannumAA or PhenoAA PLACO+ loci from their regional coverage limitation. In the integrated evidence dot-map, these strong colocalization rows appear as the highest-confidence locus-evidence component rather than as proof of directional causality (Figure 4). The lead variants, chromosome locations, regional SNP counts, and PP.H_4_ values for the six strong colocalization signals are listed in Table 4.

GRCh37 positional annotation clarified the context of the six strong colocalization lead variants. rs56239981 overlapped the noncoding transcript *LINC03178*, with *GRB14* as the nearest protein-coding gene; rs12548566 overlapped *ZFHX4-AS1*, with *ZFHX4* as the nearest protein-coding gene. The remaining loci overlapped *RCAN1*, *R3HDM1*, *ZNF778*, or the release-dependent *LINC01169*/*RP11-321F6.1* locus (Table 4). These labels describe positional context and do not identify causal target genes.

### 3.8. Multivariable MR Mechanism-Oriented Sensitivity Analysis

**Figure 5:**
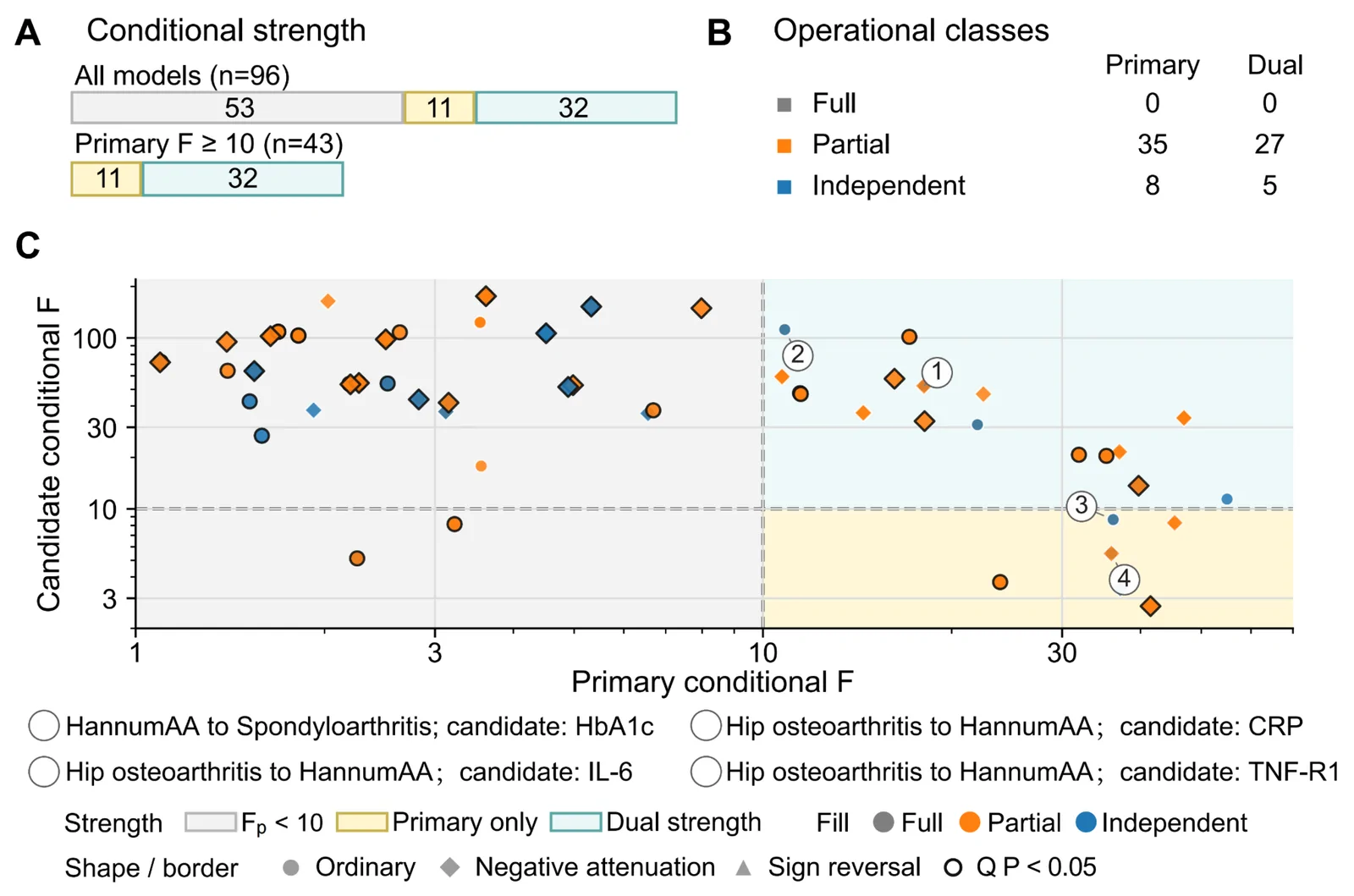
Conditional instrument strength and operational effect-pattern summary for MVMR. Panel (**A**) summarizes the 96 models by conditional-strength stratum: 53 had primary-exposure conditional *F* < 10, 11 had primary-exposure *F* ≥ 10 with candidate-trait *F* < 10, and 32 had dual *F* ≥ 10. Panel (**B**) shows the operational class counts for the primary-strength and dual-strength subsets: fully attenuated, 0/0; partially attenuated, 35/27; and independent signal, 8/5. Panel (**C**) plots primary-exposure conditional *F* against candidate-trait conditional *F*. Numbered models are: 1, HannumAA to spondyloarthritis adjusted for HbA1c; 2, hip osteoarthritis to HannumAA adjusted for CRP; 3, hip osteoarthritis to HannumAA adjusted for IL-6; and 4, hip osteoarthritis to HannumAA adjusted for TNF-R1. Raw MVMR *p* < 0.05 occurred in 4/43 primary-strength and 2/32 dual-strength models; none survived BH-FDR or Bonferroni correction. Point color indicates the operational class, marker shape indicates ordinary, negative attenuation, or sign-reversal behavior, and a dark outline denotes nominal residual heterogeneity. These operational labels describe effect-magnitude change and do not establish mediation, mechanism-specific causality, biological independence, or statistical significance of the adjusted effect.

All 96 MVMR models were reconstructed from their model-specific allele-valid SNP matrices. Forty-three models had primary-exposure conditional *F* ≥ 10, and 32 of these also had candidate-exposure conditional *F* ≥ 10; the remaining 11 primary-strength models retained candidate-exposure conditional *F* < 10. Using exact-SNP matched univariable comparators changed the assigned class in 48 of 96 models and 17 of 43 primary-strength models. The conditional-strength distribution and operational class summary are shown in Figure 5.

Across all 96 models, the operational counts were zero fully attenuated, 73 partially attenuated, and 23 independent signal. The 43 primary-strength models comprised zero fully attenuated, 35 partially attenuated, and eight independent-signal models; the 32 dual-strength models comprised zero, 27, and five models, respectively. These labels summarize prespecified changes in effect magnitude and do not estimate mediation or establish biological independence.

Adjusted absolute effect magnitude increased in 19 of 43 primary-strength models, with no sign reversal. Four primary-strength adjusted estimates had nominal *p* < 0.05, but none survived Bonferroni or false-discovery-rate correction; the dual-strength subset contained two nominal associations, also with none surviving correction. Residual *Q* heterogeneity was nominal in 10 of 43 primary-strength models and remained significant after false-discovery-rate correction in three. These findings, together with unavailable cross-trait covariance information, support use of MVMR as a descriptive mechanism-sensitivity analysis. The complete mechanism matrix and exact-SNP matched paired-effect profiles are shown in Supplementary Figures S11 and S12, respectively.

### 3.9. Integrated Evidence Synthesis

The integrated evidence synthesis combined directional MR, genome-wide genetic correlation, PLACO+ locus burden, colocalization, and strength-restricted exact-SNP-matched MVMR summaries across all 80 EAA–musculoskeletal rows. No row simultaneously combined a multiplicity-robust directional MR association, FDR-significant genetic correlation or strong colocalization, and a multiplicity-robust adjusted MVMR association. The matrix should therefore be interpreted as a comparative visualization of complementary evidence layers rather than a causal-ranking system.

**Figure 6:**
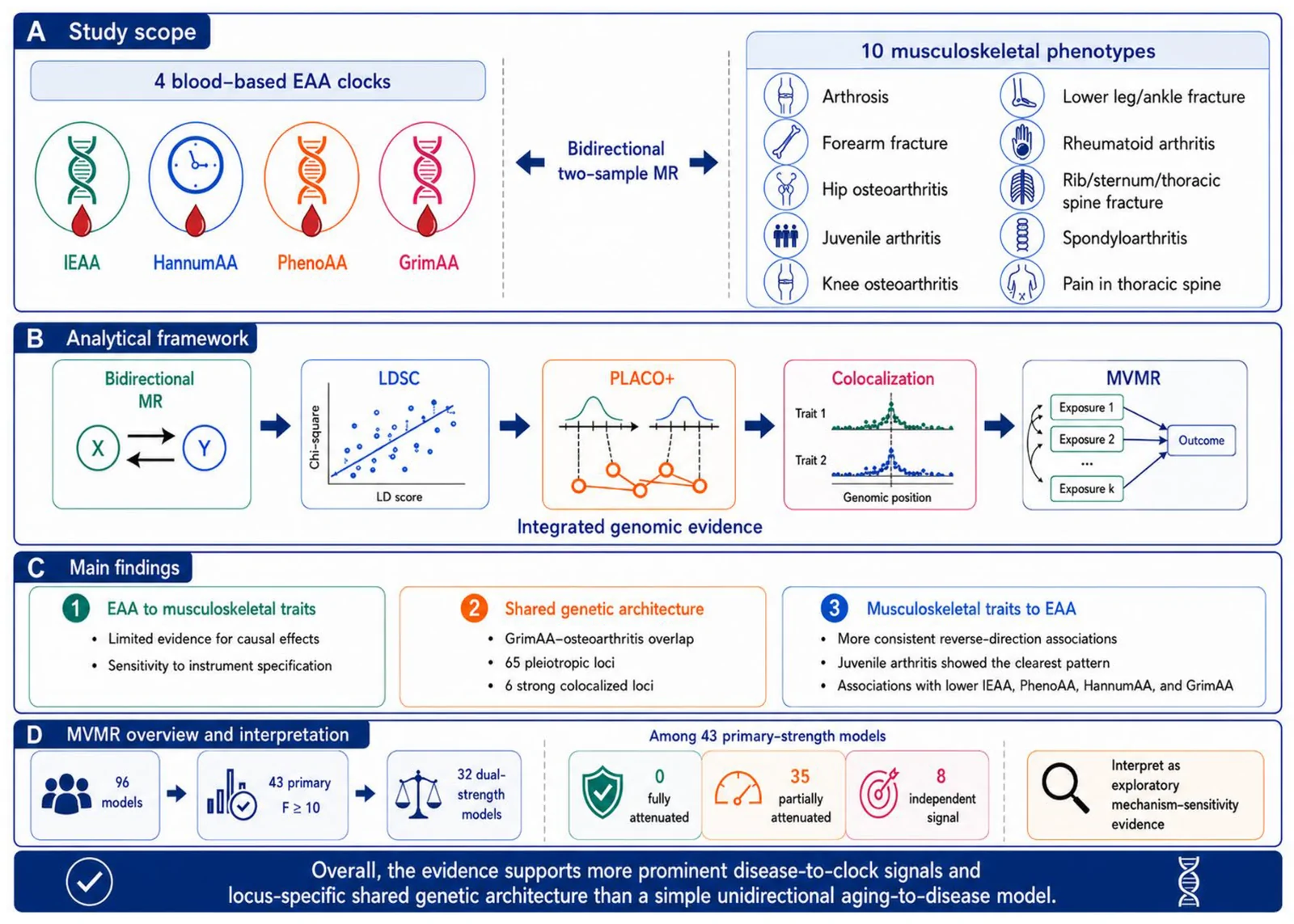
Concise summary of the study scope, analytical framework, and principal findings. Panel (**A**) shows the four blood-based epigenetic age acceleration clocks and ten musculoskeletal phenotypes evaluated by bidirectional two-sample MR. Panel (**B**) summarizes the integrated analytical framework comprising bidirectional MR, LDSC, PLACO+, colocalization, and MVMR. Panel (**C**) summarizes the directional asymmetry and the principal shared-genetic-architecture findings. Panel (**D**) shows conditional-instrument-strength restriction and the operational MVMR classifications among the 43 primary-strength models. The MVMR labels describe prespecified changes in effect magnitude and do not establish mediation or biological independence.

The integrated evidence map highlighted rows driven mainly by high-confidence locus evidence rather than nominal MR *p*-values, including GrimAA–hip osteoarthritis, GrimAA–arthrosis, and IEAA–forearm fracture. Dot size and opacity are column-normalized for display, so the visualization should be used to compare relative intensity within each evidence component, not as a single absolute evidence score across columns. The full 80-row integrated evidence matrix is provided in Supplementary Table S9. The study scope, analytical framework, and principal evidence pattern are summarized in Figure 6.

## 4. Discussion

This study indicates that the relationship between epigenetic aging and musculoskeletal disease is not adequately described by a single causal pathway. The primary MR results were directionally asymmetric: genetically predicted EAA showed limited evidence for broad effects on musculoskeletal liability, whereas several musculoskeletal liabilities were associated with EAA outcomes. This asymmetry is biologically plausible. Musculoskeletal disease can generate chronic inflammatory tone, altered immune-cell composition, pain-related neuroendocrine stress, reduced mobility, metabolic change, and treatment exposure, all of which may be reflected in blood-based methylation clocks [15,20,21,26,27]. The findings therefore support a model in which EAA partly captures systemic consequences or correlates of musculoskeletal disease biology, while only a subset of clock-to-disease relationships show evidence compatible with upstream genetic influence.

The clock-specific pattern is important for interpretation [16,17,18,19]. Degenerative phenotypes, particularly arthrosis and hip osteoarthritis, tended to map to PhenoAA and HannumAA in reverse MR, whereas the clearest genome-wide shared architecture involved GrimAA with hip and knee osteoarthritis. PhenoAA and GrimAA are designed to capture broader physiological aging and healthspan-related variation, whereas HannumAA is a blood-derived clock. The concentration of osteoarthritis-related signals in these clocks suggests that degenerative joint disease may be connected to systemic aging biology through inflammatory, metabolic, vascular, and tissue-repair pathways rather than through a universal acceleration of all methylation clocks [8,9,59,60,61].

All EAA outcomes in this analysis were derived from peripheral blood. The observed associations are therefore most directly interpretable as links to systemic and hematopoietic aging rather than direct evidence of aging within cartilage, subchondral bone, skeletal muscle, or synovium [15,23]. Blood-based clocks remain relevant because circulating immune, inflammatory, metabolic, and smoking-related processes contribute to musculoskeletal disease; however, they cannot substitute for tissue-specific methylation clocks or local tissue assays, and cross-tissue concordance should not be assumed.

Interpretation of the EAA instruments requires caution because the variants proxy inherited variation in composite blood-derived clock residuals rather than an experimental manipulation of methylation age. The GRCh37 annotation and direct-plus-proxy external-association screen showed that several instruments also indexed blood-cell, immune, metabolic, smoking-related, telomere, protein-biomarker, or musculoskeletal phenotypes [47,48]. Twenty-five instruments were therefore removed in the conservative Set C analysis, yet this filtering cannot establish that the retained variants act exclusively through EAA. The inverse HannumAA–spondyloarthritis estimate remained directionally consistent under the three-instrument definitions, whereas the HannumAA–pain-in-thoracic-spine and GrimAA–hip-osteoarthritis findings were sensitive to filtering. GrimAA estimates were additionally sensitive to restriction from the fallback-inclusive set to the four genome-wide-significant instruments. These analyses strengthen the transparency of the instrument assumptions while leaving horizontal pleiotropy and construct complexity unresolved.

The LDSC results provide the strongest evidence for diffuse shared genetic architecture, with GrimAA genetically correlated with both hip osteoarthritis and knee osteoarthritis at FDR significance. These correlations do not establish causal direction, but they indicate that alleles influencing GrimAA also tend to influence osteoarthritis susceptibility [34,35]. Clinically, this pattern is consistent with osteoarthritis being more than a purely local mechanical disease [1,8,9,62]. Hip and knee osteoarthritis often coexist with obesity, reduced mobility, multimorbidity, chronic pain, and systemic low-grade inflammation; GrimAA may capture genetic liability related to these broader healthspan dimensions [19]. Because the strongest LDSC signal did not coincide with a fully concordant directional MR and MVMR pattern, GrimAA should not be interpreted as a direct causal driver of osteoarthritis on the basis of these data alone; nevertheless, the genetic correlation justifies prioritizing the GrimAA–osteoarthritis axis for replication and mechanistic follow-up.

The reverse-MR pattern for juvenile arthritis requires disease-specific interpretation. Juvenile-arthritis liability was associated with lower IEAA, PhenoAA, HannumAA, and GrimAA, but this direction should not be interpreted as protection against biological aging. The phenotype represents pediatric-onset immune disease, whereas the EAA outcomes were derived largely from adult blood-based clocks [15,23]. Developmental timing, leukocyte-composition shifts, immune activation, ascertainment, and treatment-related biology could therefore produce inverse blood-methylation signals that do not correspond to slower organismal aging [63,64,65,66]. The exact three-SNP instruments were strong by conventional *F*-statistic criteria, and Steiger comparisons supported the tested direction over the examined prevalence range [49]. However, the exposure GWAS contained only 788 cases, the effective case–control sample size was approximately 3138, and the later overlapping FinnGen release showed smaller SNP-exposure coefficients. Accordingly, winner’s curse [58], discovery-sample imprecision, sparse-instrument diagnostics, and the absence of independent replication remain material limitations. The univariable *F* statistics reported here do not resolve the separate conditional weak-instrument limitations of the MVMR models.

The inflammatory arthritis findings also highlight the distinction between immune-mediated disease and degenerative joint disease [10,11,63,64,67,68,69]. Rheumatoid arthritis liability showed an inverse association with IEAA, while HannumAA signals were observed for spondyloarthritis and juvenile arthritis in the PLACO+ layer. These results do not converge on a simple acceleration model. Instead, they suggest that immune-mediated musculoskeletal traits may perturb blood-methylation profiles through immune lineage composition, cytokine exposure, antigen-presentation biology, or treatment pathways [65,66,67,70,71,72]. This interpretation is reinforced by the MHC-flanking PLACO+ signal near rs80136959, which is compatible with immune-adjacent cross-trait association but should not be treated as proof of an independent shared causal variant because of the complexity of the MHC region and non-evaluable colocalization [10,36,37,64,69,73]. Immunosuppressive treatment could modify methylation profiles in clinical disease cohorts, but treatment exposure is not identifiable in this summary–data MR framework and should not be treated as the primary explanation for the inverse estimates. The rheumatoid arthritis and juvenile-arthritis findings may also reflect immune-cell or HLA-related genetic effects that are not equivalent to generalized deceleration of biological aging.

Locus-level evidence sharpened, but also constrained, biological interpretation. The *GDF5* promoter/5′ untranslated-region variant rs143383 is a compelling candidate in osteoarticular biology because *GDF5* is involved in skeletal and joint development and has well-established relevance to joint morphology and osteoarthritis susceptibility [74,75,76,77,78]. In this study, rs143383 appeared as a PLACO+ signal for HannumAA with arthrosis and hip osteoarthritis, but colocalization for these regions was not evaluable because no harmonized regional SNPs remained. The correct conclusion is therefore not that rs143383 is a shared causal variant for HannumAA and osteoarthritis, but that it is a biologically plausible cross-trait candidate requiring improved regional coverage and fine-mapping [36,37,74].

The *ASCL1*-adjacent PhenoAA signals should be even more cautiously framed. Their occurrence in arthrosis and hip osteoarthritis is compatible with a candidate cross-trait signal, but the available evidence does not establish a direct role for *ASCL1* in osteoarthritis biology or methylation aging. Without evaluable colocalization, these loci remain prioritization signals rather than mechanistic conclusions [36,37]. This distinction is clinically important: genetic prioritization can identify regions worth follow-up, but it should not be translated into biomarker or therapeutic claims unless regional support, functional annotation, and external replication converge.

By contrast, the six strong colocalized loci provide more direct support for shared regional association models [37]. They involved GrimAA with hip osteoarthritis, GrimAA with arthrosis, and IEAA with forearm fracture, with the strongest PP.H_4_ values observed for GrimAA–hip osteoarthritis at rs56239981 and IEAA–forearm fracture at rs12548566. These findings imply that, at selected loci, the EAA and musculoskeletal association signals are compatible with a shared causal variant. Even here, colocalization is not equivalent to causal direction: the shared variant may influence both traits through a common upstream pathway, or one trait may lie upstream of the other [28,29]. The main value of these loci is to focus replication, fine-mapping, and functional annotation on regions with stronger evidence than PLACO+ alone.

The exact-SNP-matched, strength-restricted MVMR analysis materially narrows mechanistic interpretation. More than half of the models had primary-exposure conditional *F* < 10, and 11 of the 43 primary-strength models still had candidate-exposure conditional *F* < 10. Exact-SNP matching changed 17 of 43 primary-strength classifications, demonstrating that attenuation is interpretable only when the univariable comparator and adjusted model share the same instruments and outcome weights. Even after matching, the fully attenuated, partially attenuated, and independent-signal labels remain operational summaries of effect-magnitude change; they do not estimate a mediated proportion or establish pathway independence. Negative attenuation in 19 primary-strength models, residual heterogeneity in selected models, and the absence of any multiplicity-robust adjusted association further limit inference. Because cross-trait covariance and sample-overlap estimates were unavailable, gencov = 0 remained an explicit assumption. MVMR therefore contributes exploratory mechanism-sensitivity information and cannot identify a candidate trait as a mediator or mechanism-specific causal driver [38,39,40].

The clinical implications are therefore prioritization-oriented. EAA clocks should not be presented as established causal targets for preventing musculoskeletal disease on the basis of these data alone [15,28,29]. Instead, clock-specific signals may help identify disease contexts in which systemic aging biology is genetically linked to musculoskeletal outcomes. GrimAA–osteoarthritis relationships are the strongest candidates for studies of healthspan, multimorbidity, and degenerative joint disease [1,19,59,60,61,62,79,80]; juvenile arthritis and other immune-mediated signals warrant immune-cell-aware methylation analyses [63,64,65,66]; and fracture-related findings require attention to bone density, falls, frailty, and tissue repair [3,5,6,7,81]. Longitudinal cohorts with repeated methylation, imaging, medication, inflammatory biomarker, and disease-severity data will be needed before these genetic prioritization signals can inform clinical risk stratification [15,82].

Several limitations should shape interpretation. First, the 80-test MR matrix creates substantial multiple-testing and winner’s-curse concerns, so nominal associations require conservative framing. Second, public GWAS phenotypes lack detailed severity, duration, age at onset, imaging, treatment, pain, and functional information. Third, all EAA outcomes were blood-derived and cannot be assumed to represent methylation aging within cartilage, subchondral bone, skeletal muscle, or synovium. Fourth, external-association screening depends on database coverage, phenotype definitions, reporting thresholds, and European proxy representation and cannot eliminate horizontal pleiotropy [48]. Fifth, 28 PLACO+ loci were not evaluable by colocalization because regional harmonization failed. Sixth, the juvenile-arthritis analysis was limited by 788 cases, sparse instruments, approximate power calculations based on reported overall EAA GWAS sample sizes, and possible winner’s curse. Seventh, 53 of 96 MVMR models had primary conditional *F* < 10, 11 of the remaining 43 had candidate-exposure conditional *F* < 10, residual heterogeneity persisted, and the exposure–covariance term could not be estimated [40]. Finally, the analyses used predominantly European-ancestry summary statistics, limiting generalizability.

Taken together, the results support a layered interpretation: musculoskeletal disease liability appears more consistently related to EAA than EAA is to musculoskeletal disease liability; GrimAA shares genome-wide genetic architecture with osteoarthritis; selected GrimAA, IEAA, and musculoskeletal loci show strong colocalization; and several PLACO+ candidate loci remain biologically interesting but unresolved because of regional coverage limitations [36,37]. This hierarchy provides a defensible basis for replication and mechanistic follow-up while avoiding overstatement of causal or translational certainty.

## 5. Conclusions

Across a ten-phenotype bidirectional MR framework, musculoskeletal disease liability mapped more richly onto EAA than EAA mapped onto musculoskeletal liability. Multi-layer genomic triangulation refined this pattern: GrimAA showed FDR-significant genetic correlation with osteoarthritis, PLACO+ identified candidate cross-trait loci, and colocalization supported six strong shared-variant models in selected GrimAA–osteoarthritis and IEAA–forearm-fracture regions. Forward MR signals were sensitive to instrument filtering and GrimAA threshold choice; juvenile-arthritis reverse estimates remained limited by the small discovery case count and potential winner’s curse; and the exact-SNP-matched MVMR results provided descriptive sensitivity patterns without multiplicity-robust adjusted associations. The evidence therefore supports a prioritized genomic map rather than a definitive causal or mechanistic hierarchy.

Overall, the evidence supports a prioritized genomic map rather than a definitive causal hierarchy. *GDF5*/rs143383, the MHC-flanking rs80136959 region, and *ASCL1*-adjacent PhenoAA loci should be reported as PLACO+ candidate associations unless improved regional coverage and colocalization support become available. The integrated evidence map and Supplementary Tables identify the most defensible signals for replication, fine-mapping, and mechanistic follow-up.

## Figures and Tables

**Table 1:** Definitions, measurement characteristics, and representative applications of the four epigenetic age acceleration measures used in this study.

Measure	Definition	Measurement	Application
IEAA	Epigenetic age acceleration from the Horvath multi-tissue clock after regressing DNAm age on chronological age and estimated blood-cell composition; positive values indicate cell-intrinsic accelerated aging [16].	Residual-based DNAm metric (Horvath clock, 353 CpGs), blood-cell-adjusted IEAA.	Used for cell-intrinsic biological aging studies independent of leukocyte-shift confounding; applied in neuroaging analyses, cognitive-decline risk modeling, and immune-senescence stratification.
HannumAA	Blood-based epigenetic age acceleration from the Hannum clock residual versus chronological age; mainly reflects hematopoietic and immune-aging patterns [17].	Residual-based DNAm metric (Hannum blood clock, 71 CpGs), typically derived in whole blood.	Used in inflammation and cardiometabolic epidemiology; associated with metabolic syndrome burden, cardiovascular-risk markers, and systemic immune-aging status.
GrimAA	Acceleration of DNAm GrimAge over chronological age; this mortality-oriented clock integrates methylation surrogates for plasma proteins and smoking pack-years [19].	Residual-based DNAm GrimAge metric (protein-surrogate and smoking-informed methylation model).	Used for mortality and healthspan prediction; strongly linked to all-cause mortality, major cardiovascular events, cancer incidence, frailty progression, and hospitalization risk.
PhenoAA	Acceleration of DNAm PhenoAge relative to chronological age; quantifies deviation in phenotypic and clinical aging burden [18].	Residual-based DNAm PhenoAge metric trained from clinical-biomarker phenotypic age and methylation profile.	Used for multimorbidity and disease-onset prediction; associated with type 2 diabetes, chronic kidney disease, cardiovascular disease, functional decline, and survival outcomes.

**Table 2:** Public GWAS datasets used in the expanded bidirectional MR analysis, including 10 musculoskeletal phenotypes and four epigenetic age acceleration traits.

GWAS ID	Trait	Number of Cases	Number of Controls	PMID
finn-b-ST19_FRACT_LOWER_LEG _INCLU_ANKLE	Lower leg/ankle fracture	10,489	191,178	36653562
finn-b-ST19_FRACT_FOREA	Forearm fracture	9956	205,768	36653562
finn-b-ST19_FRACT_RIBS _STERNUM_THORACIC_SPINE	Rib/sternum/thoracic spine fracture	4070	211,861	36653562
ebi-a-GCST007092	Hip osteoarthritis	39,427	378,169	30664745
ebi-a-GCST007090	Knee osteoarthritis	24,955	378,169	30664745
ebi-a-GCST90018910	Rheumatoid arthritis	8255	409,001	34594039
finn-b-JUVEN_ARTHR	Juvenile arthritis	788	172,834	36653562
finn-b-M13_ARTHROSIS	Arthrosis	37,233	147,221	36653562
finn-b-SPONDYLOARTHRITIS	Spondyloarthritis	3037	198,544	36653562
finn-b-M13_THORACISPINEPAIN	Pain in thoracic spine	2202	164,682	36653562
ebi-a-GCST90014302	Intrinsic epigenetic age acceleration	34,461	34,461	34187551
ebi-a-GCST90014289	DNA methylation Hannum age acceleration	34,449	34,449	34187551
ebi-a-GCST90014300	DNA methylation GrimAge acceleration	34,467	34,467	34187551
ebi-a-GCST90014292	DNA methylation PhenoAge acceleration	34,463	34,463	34187551

**Table 3:** Heterogeneity and pleiotropy diagnostics for the forward and reverse associations taken into extended sensitivity analysis.

Direction	Exposure	Outcome	MR Egger *Q*	MR Egger *p*	IVW *Q*	IVW *p*	Egger Intercept	Intercept *p*
forward	HannumAA	Spondyloarthritis	16.034	0.025	16.042	0.042	−0.004	0.957
forward	HannumAA	Pain in thoracic spine	7.397	0.389	7.471	0.487	−0.014	0.800
reverse	Rheumatoid arthritis	IEAA	15.761	0.541	19.623	0.354	0.046	0.066
reverse	Arthrosis	HannumAA	28.259	0.003	28.278	0.005	0.003	0.933
reverse	Arthrosis	PhenoAA	7.830	0.728	9.551	0.655	−0.045	0.216
reverse	Juvenile arthritis	GrimAA	4.177	0.041	4.202	0.122	0.026	0.950
reverse	Juvenile arthritis	HannumAA	0.675	0.411	0.706	0.703	−0.024	0.889
reverse	Juvenile arthritis	IEAA	0.069	0.794	0.994	0.608	0.174	0.512
reverse	Juvenile arthritis	PhenoAA	1.514	0.219	1.514	0.469	−0.004	0.989
reverse	Hip osteoarthritis	HannumAA	21.875	0.528	24.072	0.458	−0.070	0.152
reverse	Hip osteoarthritis	PhenoAA	18.792	0.713	18.867	0.759	−0.017	0.787

**Table 4:** Strong colocalization loci and GRCh37 positional gene annotations. PP.*H*_4_ is the posterior probability for hypothesis *H*_4_, under which both traits are associated and share a causal variant within the analyzed region in the coloc framework. Genes are positional annotations based on GRCh37 transcript overlap or nearest-gene mapping and do not establish the causal target gene. Distances for nearest protein-coding genes are measured to the gene body. For rs118008484, Ensembl GRCh37 GTF annotation 87 and the current HGNC record provide different biotype classifications for the *LINC01169*/*RP11-321F6.1* locus; both source-specific annotations are retained without inferring function.

EAA Clock	Musculoskeletal Trait	Lead SNP	Positional Gene Annotation	Chr	No. SNPs	PP.*H*_4_
GrimAA	Hip osteoarthritis	rs56239981	Overlapping: *LINC03178* (lincRNA); nearest protein-coding: *GRB14* (167.1 kb from gene body)	2	749	0.919
IEAA	Forearm fracture	rs12548566	Overlapping: *ZFHX4-AS1* (antisense); nearest protein-coding: *ZFHX4* (132.4 kb from gene body)	8	1042	0.918
GrimAA	Arthrosis	rs2251958	*RCAN1* (protein-coding)	21	879	0.805
GrimAA	Arthrosis	rs75936851	*R3HDM1* (protein-coding)	2	625	0.803
GrimAA	Hip osteoarthritis	rs72819395	*ZNF778* (protein-coding)	16	1127	0.801
IEAA	Forearm fracture	rs118008484	*LINC01169* (GRCh37 GTF 87: protein-coding; current HGNC: long non-coding RNA)	15	1440	0.786

## Data Availability

All GWAS summary statistics used in this study were obtained from publicly available resources, primarily the IEU OpenGWAS platform and FinnGen, with identifiers listed in Table 2.

## Supplementary Materials

The following supporting information can be downloaded at: https://www.mdpi.com/article/10.3390/genes17080878/s1, Supplementary Figure S1, forward-direction sensitivity profiles for the two HannumAA associations; Supplementary Figure S2, juvenile-arthritis reverse-MR sensitivity profiles using the resolved three-SNP instrument set; Supplementary Figure S3, additional reverse-direction MR sensitivity profiles; Supplementary Figure S4, EAA-instrument annotation and external-association evidence atlas; Supplementary Figure S5, Set A/B/C robustness of forward EAA associations; Supplementary Figure S6, GrimAA instrument-threshold sensitivity; Supplementary Figure S7, juvenile-arthritis instrument strength, directionality, and detectability; Supplementary Figure S8, LDSC genetic-correlation heatmap; Supplementary Figure S9, PLACO+ locus-burden summary; Supplementary Figure S10, colocalization summary; Supplementary Figure S11, MVMR mechanism matrix; and Supplementary Figure S12, exact-SNP matched univariable and MVMR-adjusted effect profiles. Supplementary Tables S1–S13 are provided in a unified workbook: S1, EAA instruments; S2, forward primary MR; S3, reverse primary MR; S4, LDSC genetic correlations; S5, PLACO+ trait-pair summary; S6, independent PLACO+ loci; S7, colocalization results; S8, model-specific MVMR results; S9, integrated evidence synthesis; S10, EAA-instrument biological interpretation and Set C decisions; S11, Set A/B/C forward-MR robustness; S12, juvenile-arthritis instrument-strength audit; and S13, liability-scale Steiger and minimum-detectable-effect analyses.
