# Supplementary material for: Shared Genetic Architecture Between Epigenetic Aging and Musculoskeletal Diseases
Source: Genes (Basel). 2026 Jul 28;17(8):878. doi: 10.3390/genes17080878 (PMC13512585; doi:10.3390/genes17080878)
Supplement: Supplementary file 1 [file genes-17-00878-s001.zip › Supplementary_Figure_Legends_S1-S12.pdf]

Supplementary Figure Legends

Supplementary Figure S1

Forward-direction MR sensitivity profiles for the two nominal HannumAA associations. Panel A shows the effect of HannumAA on pain in thoracic spine, and Panel B shows the effect of HannumAA on spondyloarthritis. For each association, the scatter plot compares SNP-exposure and SNP-outcome effects with regression lines for the available MR estimators, and the leave-one-out plot shows the IVW estimate after sequential removal of each instrument. These panels should be interpreted together with the heterogeneity and MR-Egger-intercept diagnostics in Table 3.

Supplementary Figure S2

Juvenile-arthritis reverse-MR sensitivity profiles using the resolved three-SNP primary instrument set. Panels A–D show the effects of juvenile arthritis on IEAA, PhenoAA, HannumAA, and GrimAA, respectively. Within each panel, the scatter plot shows harmonized SNP-exposure and SNP-outcome effects and the available MR estimator lines; the leave-one-out plot shows IVW estimates and 95% confidence intervals after sequential omission of each instrument. All calculations use rs115681000, rs116080026, and rs9266804. rs2073722 was excluded during harmonization because of incompatible alleles. Because only three instruments were available, MR-Egger and mode estimates are presented as descriptive sensitivity analyses.

Supplementary Figure S3

Additional reverse-direction MR sensitivity profiles. Panels A and B show arthrosis to PhenoAA and HannumAA, Panel C shows rheumatoid arthritis to IEAA, and Panels D and E show hip osteoarthritis to HannumAA and PhenoAA. Each association is represented by an SNP-level scatter plot and an IVW leave-one-out plot. Visual direction was evaluated jointly with the numeric heterogeneity and pleiotropy diagnostics and was not treated as evidence of robustness by itself.

Supplementary Figure S4

EAA-instrument annotation and external-association evidence atlas. Rows represent the 61 instruments grouped by EAA clock. The tracks summarize the GRCh37 VEP consequence, instrument-selection threshold, SNP-level F statistic, genome-wide-significant external-association domains identified for the index variant or European LD proxies, pair-specific Steiger retention, and retention in the conservative Set C reconstruction. External phenotype domains are non-mutually exclusive. Positional gene labels and phenotype-domain assignments support instrument interpretation but do not identify causal genes or exclude horizontal pleiotropy.

Supplementary Figure S5

Set A/B/C robustness of forward EAA associations. Panels correspond to GrimAA, HannumAA, IEAA, and PhenoAA. Points and horizontal lines show odds ratios and 95% confidence intervals for the original instruments (Set A), pair-specific Steiger-filtered instruments (Set B), and Steiger plus high-confidence external-association-filtered instruments (Set C). The right-hand entries show the numbers of instruments in Sets A, B, and C. Set C is a conservative sensitivity analysis and does not replace the primary instrument definition.

Supplementary Figure S6

GrimAA instrument-selection-threshold sensitivity. The fallback-inclusive pool contains all 21 selected GrimAA instruments, comprising four genome-wide-significant instruments and 17 instruments selected at the fallback threshold. The GWS-only comparison is restricted to the four genome-wide-significant instruments. Points show odds ratios and 95% confidence intervals for Sets A, B, and C; gray segments link estimates for the same musculoskeletal outcome and instrument set across threshold definitions. Outcome- and set-specific SNP counts can be lower after harmonization and filtering.

Supplementary Figure S7

Juvenile-arthritis reverse-MR instrument strength, directionality, and detectability. Panel A shows the four genome-wide-significant candidate variants, identifies the three variants retained in the primary MR, and marks F-statistic reference values of 10 and 20. Panel B shows the ratio of juvenile-arthritis liability-scale variance explained to continuous EAA-outcome variance explained across the examined prevalence assumptions; ratios above 1 support the tested juvenile arthritis-to-EAA direction under the stated assumption. Panel C shows prospective two-sided power across a prespecified absolute causal-effect grid at $\alpha = 0.05$ and $\alpha = 0.0125$. The power curves describe model-based detectability and are not post hoc power calculations based on the observed estimates.

Supplementary Figure S8

LDSC genetic correlations between EAA clocks and musculoskeletal phenotypes. The matrix displays genetic-correlation estimates for all 40 EAA-musculoskeletal pairs. Color encodes the direction and magnitude of the genetic correlation, and the plotted point size reflects statistical evidence. Asterisks identify correlations that remained significant after false-discovery-rate correction. The strongest corrected signals involved GrimAA with hip and knee osteoarthritis.

Supplementary Figure S9

PLACO+ pleiotropic-locus burden across prioritized EAA-musculoskeletal pairs. Trait pairs are ranked by the number of independent PLACO+ loci. Labels identify the leading cross-trait variant and its PLACO+ P value for each pair. These signals prioritize shared genomic regions but do not distinguish a shared causal variant from linkage or nearby independent associations.

Supplementary Figure S10

Colocalization summary for independent PLACO+ loci. Panel A ranks evaluable loci by colocalization evidence and identifies the corresponding EAA-musculoskeletal pair and lead variant. Panel B summarizes the numbers of strong, moderate, no-evidence, and non-evaluable loci. Strong evidence was defined as $PP.H_4 \geq 0.75$. Non-evaluable loci contained insufficient harmonized regional variants and were treated as coverage failures rather than biological nulls.

Supplementary Figure S11

MVMR mechanism matrix across 96 models. Rows represent 12 directional EAA-musculoskeletal pairs and columns represent eight candidate mechanism traits. Fill color denotes the retained operational class: fully attenuated, partially attenuated, or independent signal. Marker area denotes the absolute attenuation magnitude calculated from the MVMR primary-exposure estimate and the univariable estimate obtained

from the identical SNP set, capped at 100% for display. Circles denote ordinary effect changes, diamonds denote negative attenuation, and triangles denote sign reversal. Marker borders distinguish primary conditional $F < 10$, primary $F \geq 10$ with candidate-trait $F < 10$, and dual $F \geq 10$. The classes are operational descriptions and do not establish mediation, mechanism-specific causality, or biological independence.

Supplementary Figure S12

Exact-SNP matched univariable and MVMR-adjusted effect profiles for the primary-strength models. Panel A presents forward models with binary musculoskeletal outcomes. Panels B1 and B2 present reverse degenerative and inflammatory models, and Panels C1 and C2 present reverse juvenile-arthritis and fracture models. Open circles and horizontal lines show exact-SNP matched univariable estimates and 95% confidence intervals; filled triangles and horizontal lines show MVMR-adjusted estimates and 95% confidence intervals. Primary F and mechanism F are the corresponding conditional F statistics. M identifies mechanism conditional $F < 10$, Q identifies nominal residual heterogeneity, A identifies negative attenuation, and S identifies sign reversal. The operational classes do not imply mediation, biological independence, or statistical significance of the adjusted estimate.
