# Supplementary material for: Shared Genetic Architecture Between Epigenetic Aging and Musculoskeletal Diseases
Source: Genes (Basel). 2026 Jul 28;17(8):878. doi: 10.3390/genes17080878 (PMC13512585; doi:10.3390/genes17080878)
Supplement: Supplementary file 1 [file genes-17-00878-s001.zip › supplementary_figure_S10_revised1.pdf]

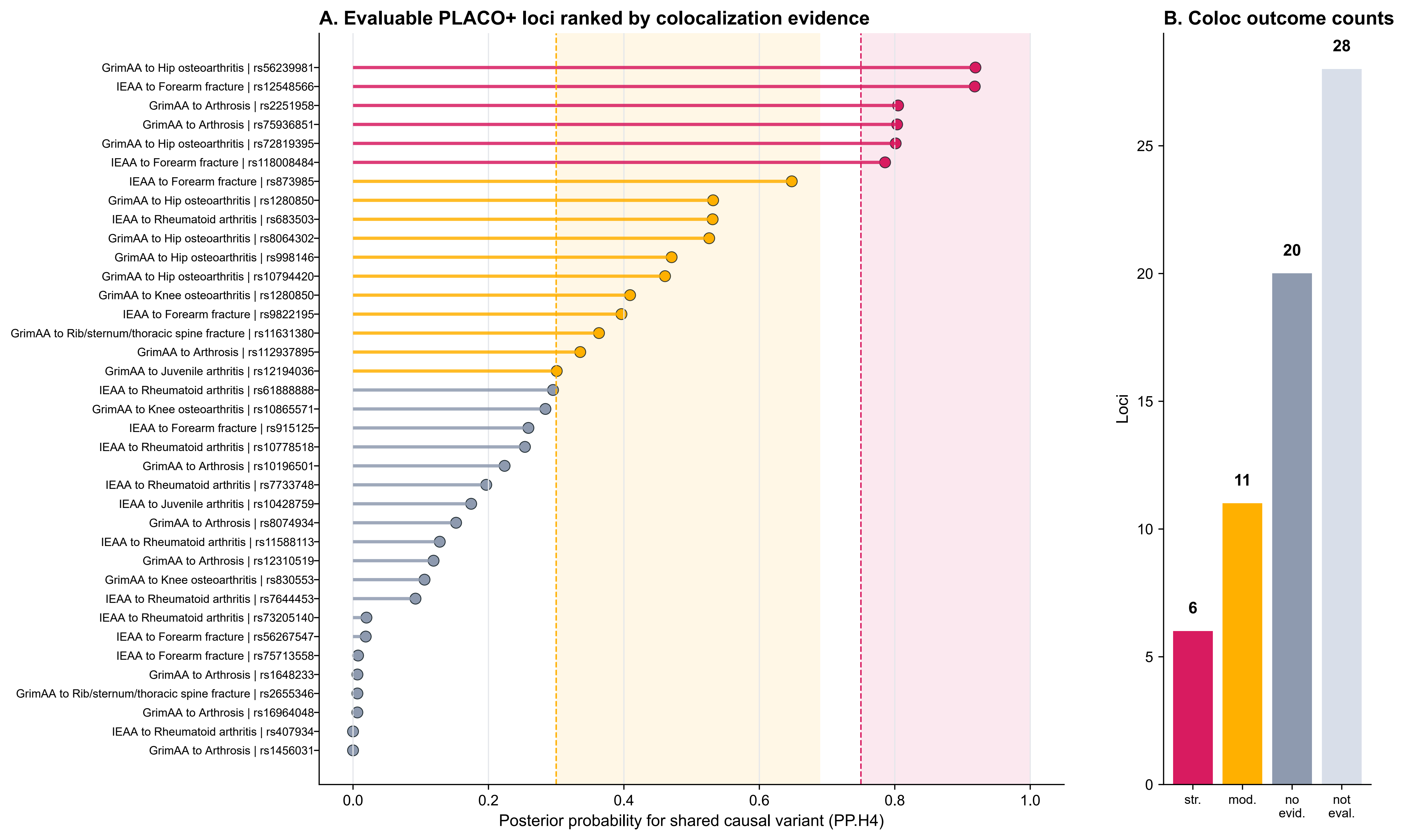

A. Evaluable PLACO+ loci ranked by colocalization evidence
GrimAA to Hip osteoarthritis | rs56239981
IEAA to Forearm fracture | rs12548566
GrimAA to Arthrosis | rs2251958
GrimAA to Arthrosis | rs75936851
GrimAA to Hip osteoarthritis | rs72819395
IEAA to Forearm fracture | rs118008484
IEAA to Forearm fracture | rs873985
GrimAA to Hip osteoarthritis | rs1280850
IEAA to Rheumatoid arthritis | rs683503
GrimAA to Hip osteoarthritis | rs8064302
GrimAA to Hip osteoarthritis | rs998146
GrimAA to Hip osteoarthritis | rs10794420
GrimAA to Knee osteoarthritis | rs1280850
IEAA to Forearm fracture | rs9822195
GrimAA to Rib/sternum/thoracic spine fracture | rs11631380
GrimAA to Arthrosis | rs112937895
GrimAA to Juvenile arthritis | rs12194036
IEAA to Rheumatoid arthritis | rs61888888
GrimAA to Knee osteoarthritis | rs10865571
IEAA to Forearm fracture | rs915125
IEAA to Rheumatoid arthritis | rs10778518
GrimAA to Arthrosis | rs10196501
IEAA to Rheumatoid arthritis | rs7733748
IEAA to Juvenile arthritis | rs10428759
GrimAA to Arthrosis | rs8074934
IEAA to Rheumatoid arthritis | rs11588113
GrimAA to Arthrosis | rs12310519
GrimAA to Knee osteoarthritis | rs830553
IEAA to Rheumatoid arthritis | rs7644453
IEAA to Rheumatoid arthritis | rs73205140
IEAA to Forearm fracture | rs56267547
IEAA to Forearm fracture | rs75713558
GrimAA to Arthrosis | rs1648233
GrimAA to Rib/sternum/thoracic spine fracture | rs2655346
GrimAA to Arthrosis | rs16964048
IEAA to Rheumatoid arthritis | rs407934
GrimAA to Arthrosis | rs1456031
0.0
0.2
0.4
0.6
0.8
1.0
Posterior probability for shared causal variant (PP.H4)
B. Coloc outcome counts
Loci
0
5
10
15
20
25
6
11
20
28
str.
mod.
no evid.
not eval.
