# Supplementary material for: Shared Genetic Architecture Between Epigenetic Aging and Musculoskeletal Diseases
Source: Genes (Basel). 2026 Jul 28;17(8):878. doi: 10.3390/genes17080878 (PMC13512585; doi:10.3390/genes17080878)
Supplement: Supplementary file 1 [file genes-17-00878-s001.zip › supplementary_figure_S11_revised1.pdf]

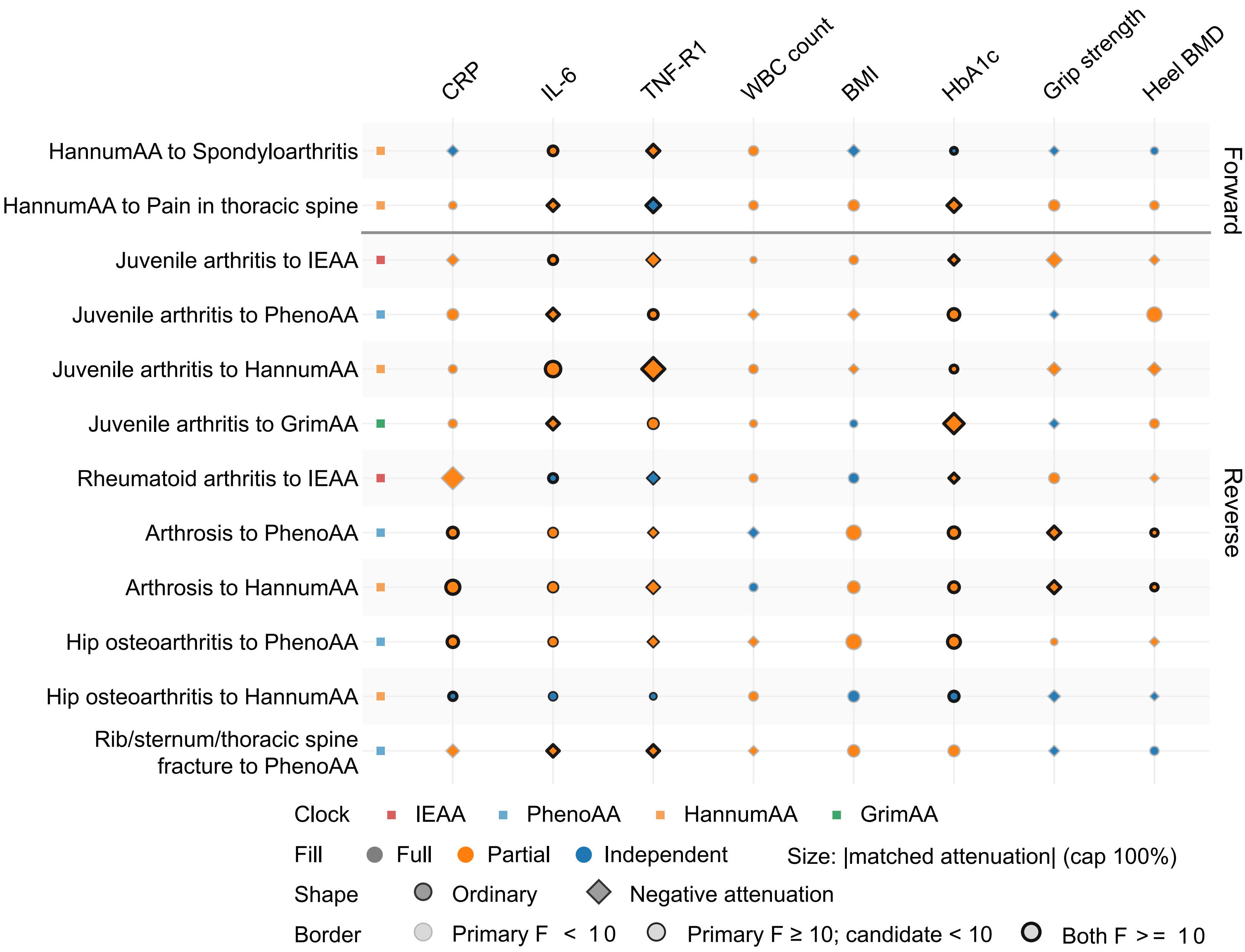

CRP
IL-6
TNF-R1
WBC count
BMI
HbA1c
Grip strength
Heel BMD
HannumAA to Spondyloarthritis
HannumAA to Pain in thoracic spine
Juvenile arthritis to IEAA
Juvenile arthritis to PhenoAA
Juvenile arthritis to HannumAA
Juvenile arthritis to GrimAA
Rheumatoid arthritis to IEAA
Arthrosis to PhenoAA
Arthrosis to HannumAA
Hip osteoarthritis to PhenoAA
Hip osteoarthritis to HannumAA
Rib/sternum/thoracic spine fracture to PhenoAA
Forward
Reverse
Clock
IEAA
PhenoAA
HannumAA
GrimAA
Fill
Full
Partial
Independent
Size: |matched attenuation| (cap 100%)
Shape
Ordinary
Negative attenuation
Border
Primary F < 10
Primary F ≥ 10; candidate < 10
Both F >= 10
