# Supplementary material for: Shared Genetic Architecture Between Epigenetic Aging and Musculoskeletal Diseases
Source: Genes (Basel). 2026 Jul 28;17(8):878. doi: 10.3390/genes17080878 (PMC13512585; doi:10.3390/genes17080878)
Supplement: Supplementary file 1 [file genes-17-00878-s001.zip › Supplementary_Figure_S12.pdf]

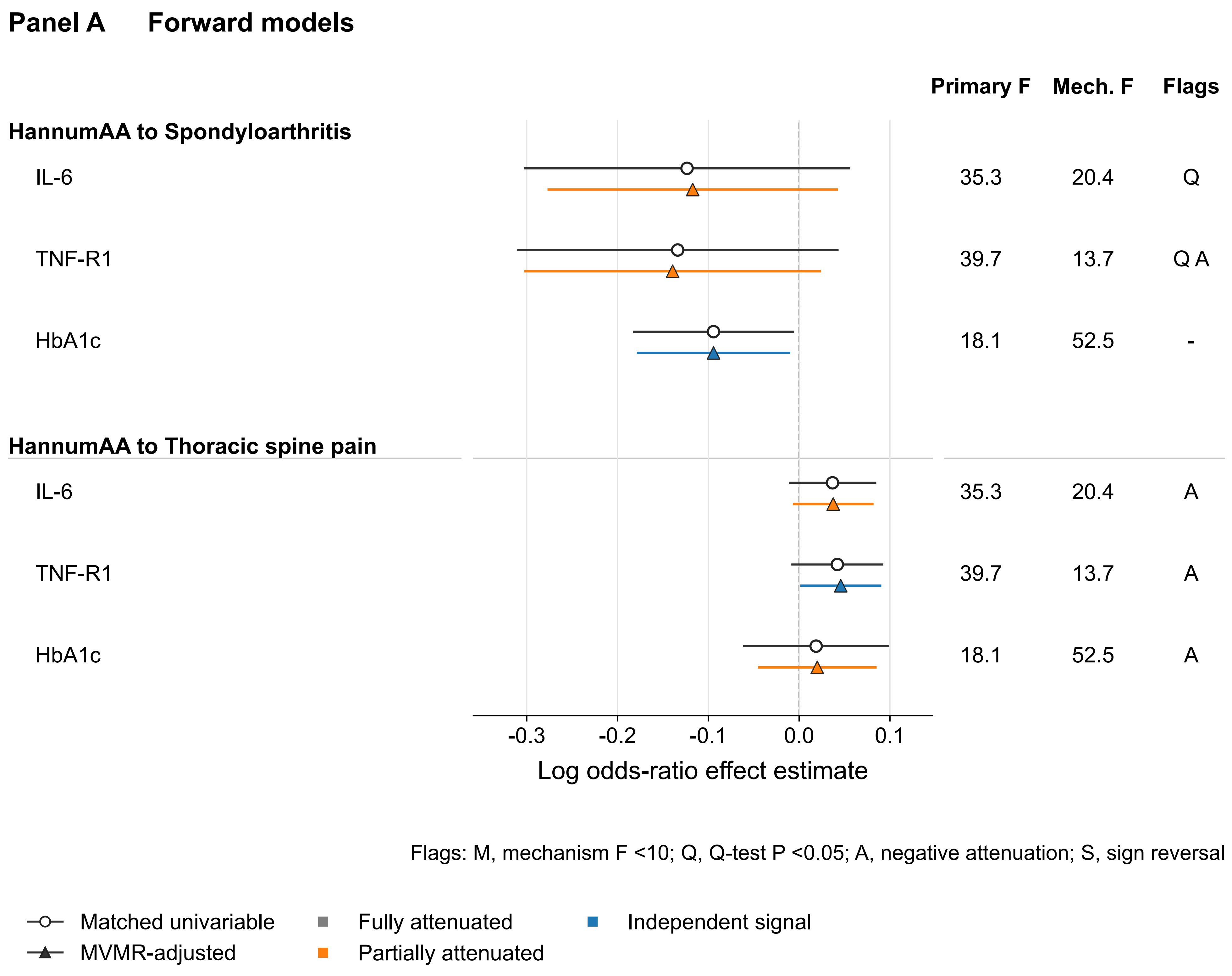
Panel A Forward models
Primary F
Mech. F
Flags
HannumAA to Spondyloarthritis
IL-6
35.3
20.4
Q
TNF-R1
39.7
13.7
Q A
HbA1c
18.1
52.5
-
HannumAA to Thoracic spine pain
IL-6
35.3
20.4
A
TNF-R1
39.7
13.7
A
HbA1c
18.1
52.5
A
-0.3
-0.2
-0.1
0.0
0.1
Log odds-ratio effect estimate
Flags: M, mechanism F <10; Q, Q-test P <0.05; A, negative attenuation; S, sign reversal
Matched univariable
MVMR-adjusted
Fully attenuated
Partially attenuated
Independent signal

Panel B1 Reverse models: arthrosis and rheumatoid arthritis

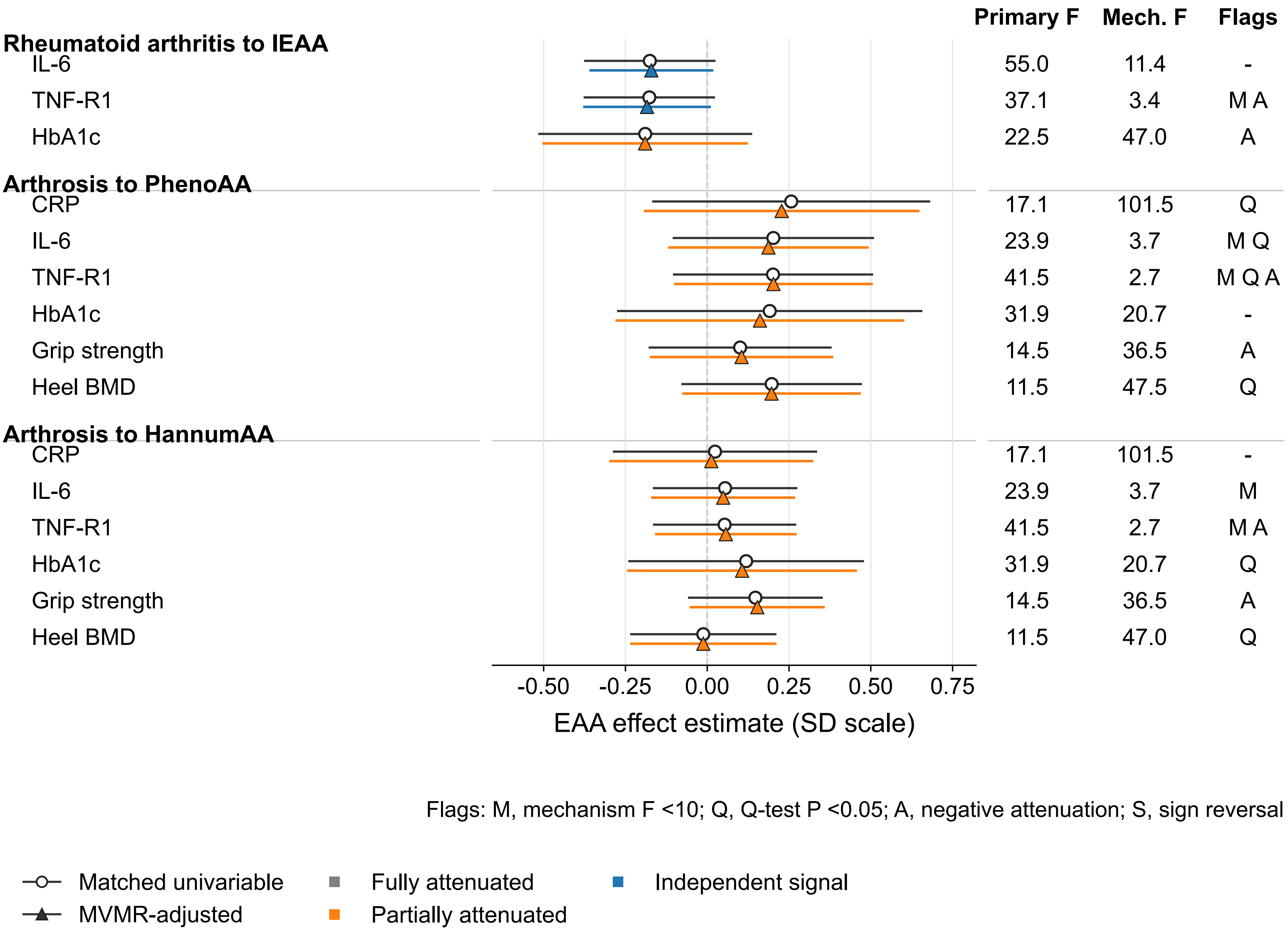

Panel B2 Reverse models: hip/knee osteoarthritis

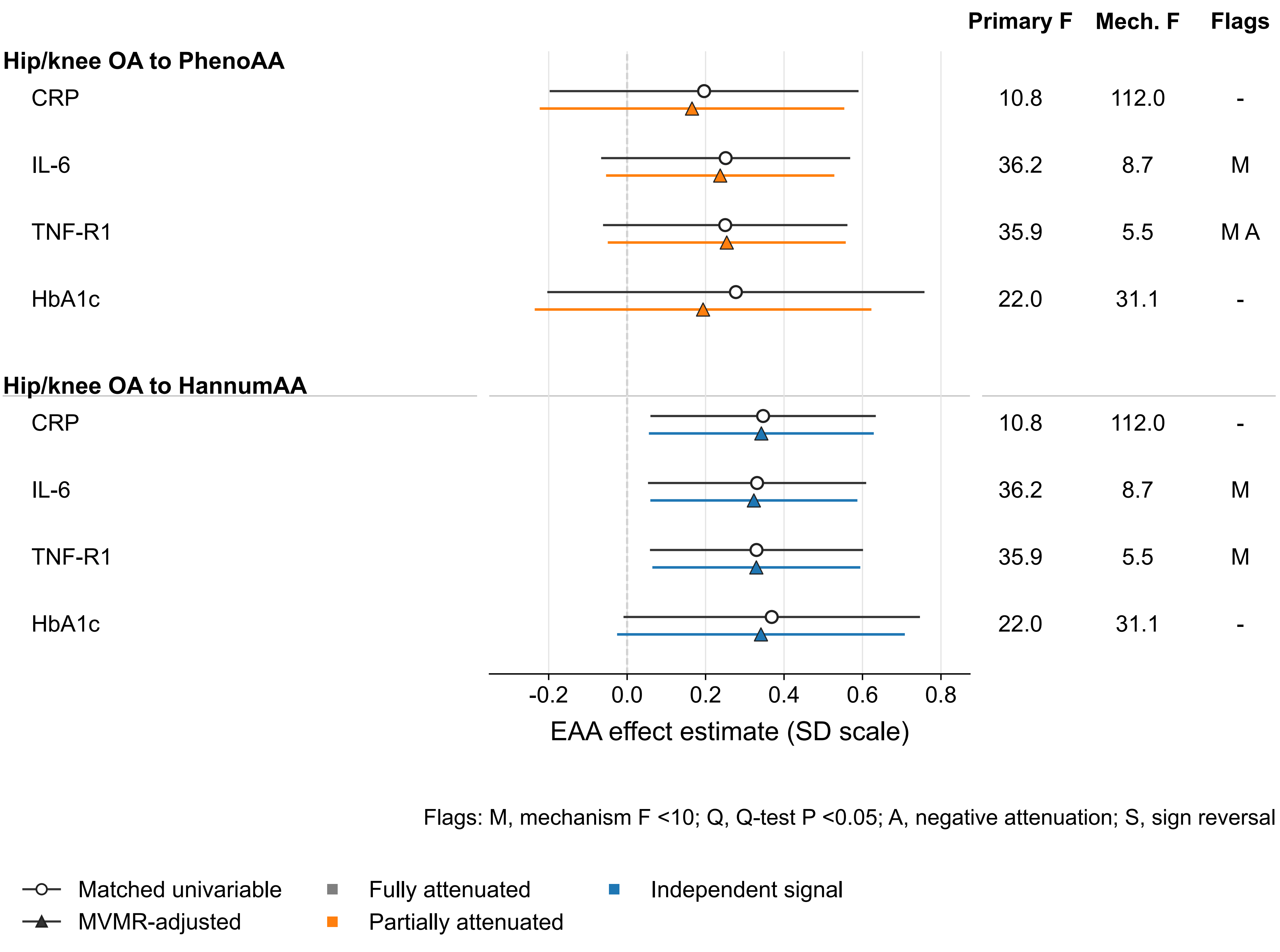

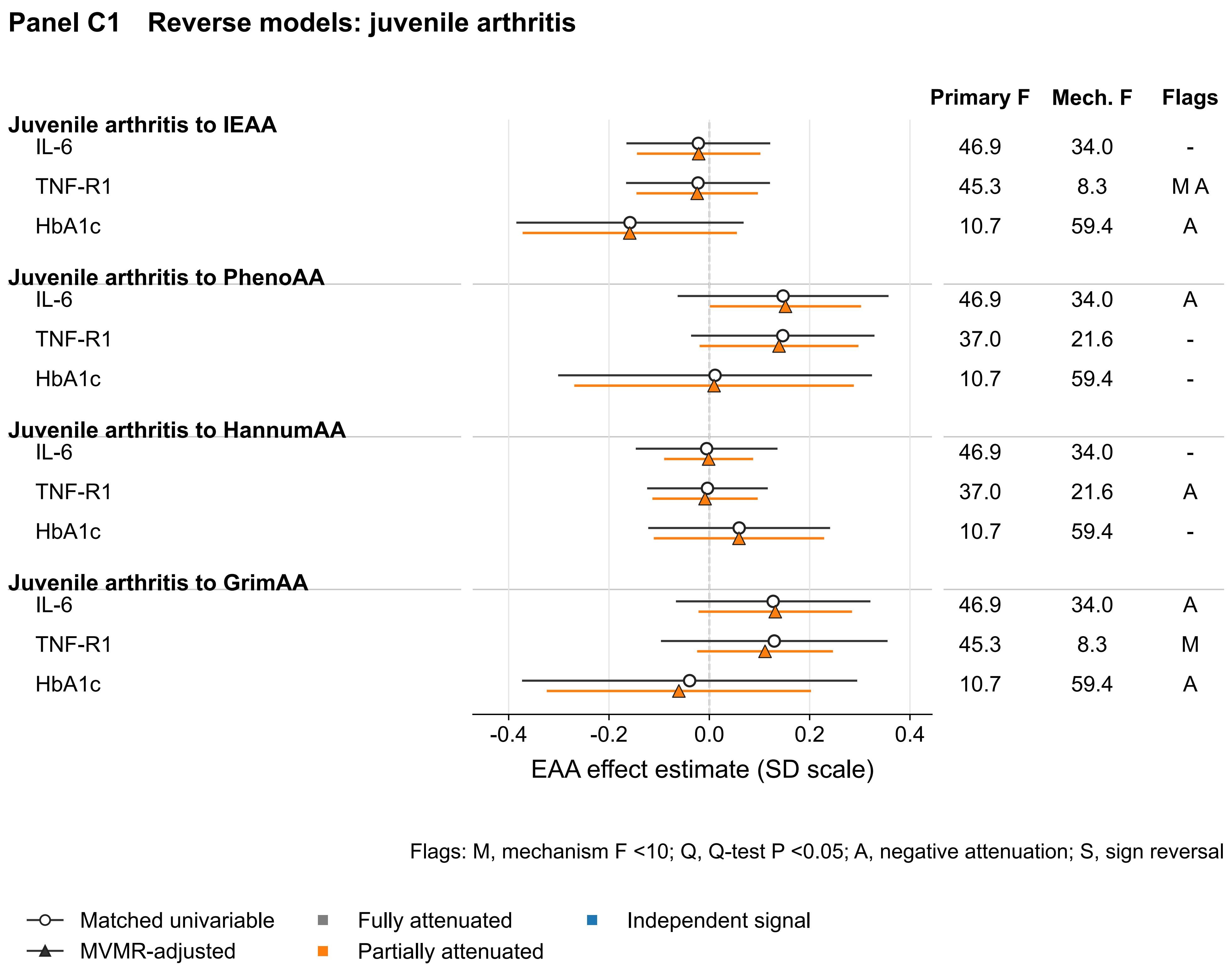
Panel C1 Reverse models: juvenile arthritis
Primary F
Mech. F
Flags
Juvenile arthritis to IEAA
IL-6 46.9 34.0 -
TNF-R1 45.3 8.3 M A
HbA1c 10.7 59.4 A
Juvenile arthritis to PhenoAA
IL-6 46.9 34.0 A
TNF-R1 37.0 21.6 -
HbA1c 10.7 59.4 -
Juvenile arthritis to HannumAA
IL-6 46.9 34.0 -
TNF-R1 37.0 21.6 A
HbA1c 10.7 59.4 -
Juvenile arthritis to GrimAA
IL-6 46.9 34.0 A
TNF-R1 45.3 8.3 M
HbA1c 10.7 59.4 A
-0.4
-0.2
0.0
0.2
0.4
EAA effect estimate (SD scale)
Flags: M, mechanism F <10; Q, Q-test P <0.05; A, negative attenuation; S, sign reversal
Matched univariable
MVMR-adjusted
Fully attenuated
Partially attenuated
Independent signal

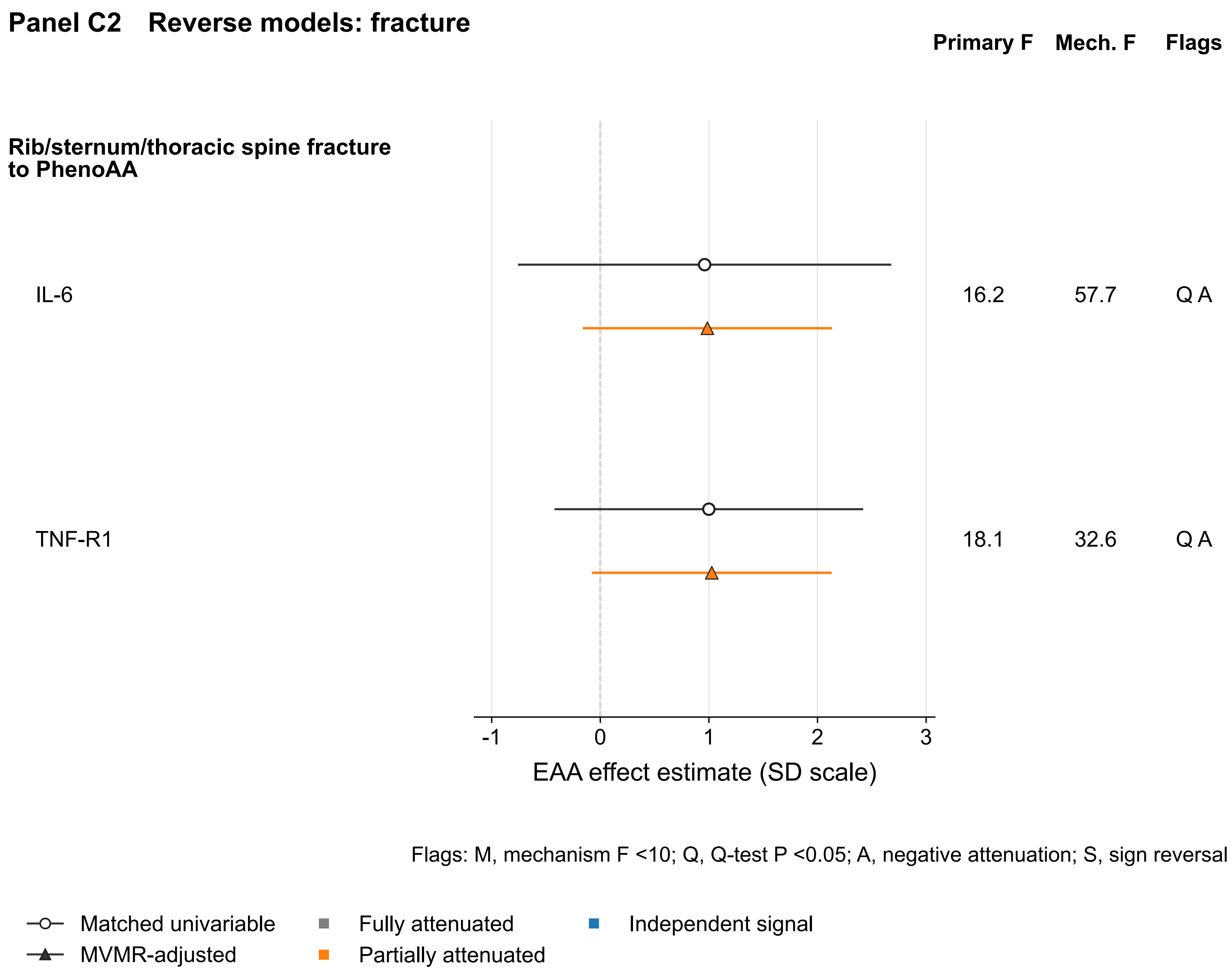
Panel C2 Reverse models: fracture
Primary F
Mech. F
Flags
Rib/sternum/thoracic spine fracture to PhenoAA
IL-6
16.2
57.7
Q A
TNF-R1
18.1
32.6
Q A
-1
0
1
2
3
EAA effect estimate (SD scale)
Flags: M, mechanism F <10; Q, Q-test P <0.05; A, negative attenuation; S, sign reversal
Matched univariable
MVMR-adjusted
Fully attenuated
Partially attenuated
Independent signal
