# Supplementary material for: Shared Genetic Architecture Between Epigenetic Aging and Musculoskeletal Diseases
Source: Genes (Basel). 2026 Jul 28;17(8):878. doi: 10.3390/genes17080878 (PMC13512585; doi:10.3390/genes17080878)
Supplement: Supplementary file 1 [file genes-17-00878-s001.zip › supplementary_figure_S1_revised1.pdf]

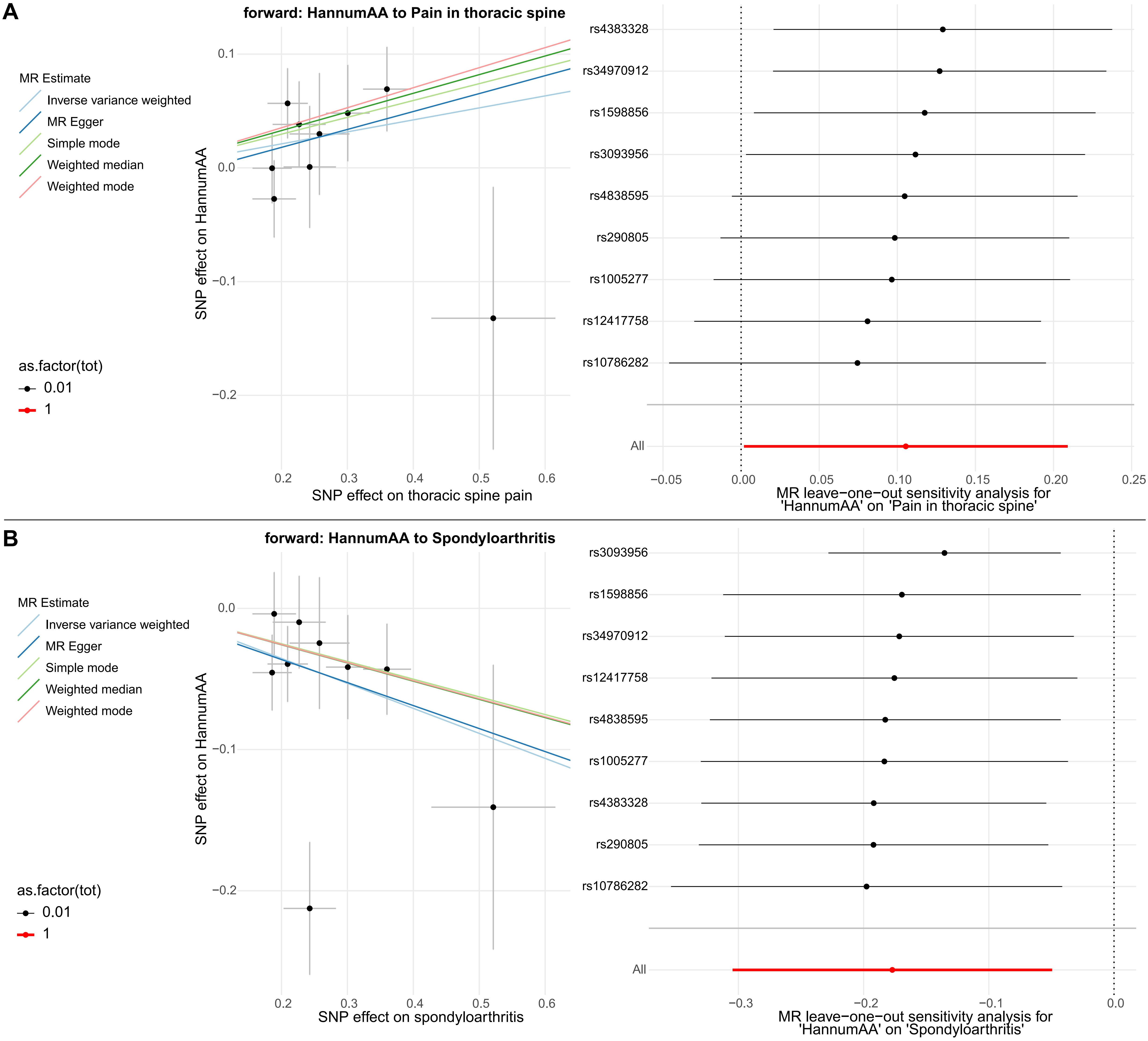
A
forward: HannumAA to Pain in thoracic spine
MR Estimate
Inverse variance weighted
MR Egger
Simple mode
Weighted median
Weighted mode
as.factor(tot)
0.01
1
SNP effect on HannumAA
SNP effect on thoracic spine pain
0.1
0.0
−0.1
−0.2
0.2
0.3
0.4
0.5
0.6
rs4383328
rs34970912
rs1598856
rs3093956
rs4838595
rs290805
rs1005277
rs12417758
rs10786282
All
−0.05
0.00
0.05
0.10
0.15
0.20
0.25
MR leave−one−out sensitivity analysis for
'HannumAA' on 'Pain in thoracic spine'
B
forward: HannumAA to Spondyloarthritis
MR Estimate
Inverse variance weighted
MR Egger
Simple mode
Weighted median
Weighted mode
as.factor(tot)
0.01
1
SNP effect on HannumAA
SNP effect on spondyloarthritis
0.0
−0.1
−0.2
0.2
0.3
0.4
0.5
0.6
rs3093956
rs1598856
rs34970912
rs12417758
rs4838595
rs1005277
rs4383328
rs290805
rs10786282
All
−0.3
−0.2
−0.1
0.0
MR leave−one−out sensitivity analysis for
'HannumAA' on 'Spondyloarthritis'
