# Supplementary material for: Shared Genetic Architecture Between Epigenetic Aging and Musculoskeletal Diseases
Source: Genes (Basel). 2026 Jul 28;17(8):878. doi: 10.3390/genes17080878 (PMC13512585; doi:10.3390/genes17080878)
Supplement: Supplementary file 1 [file genes-17-00878-s001.zip › supplementary_figure_S2_revised1.pdf]

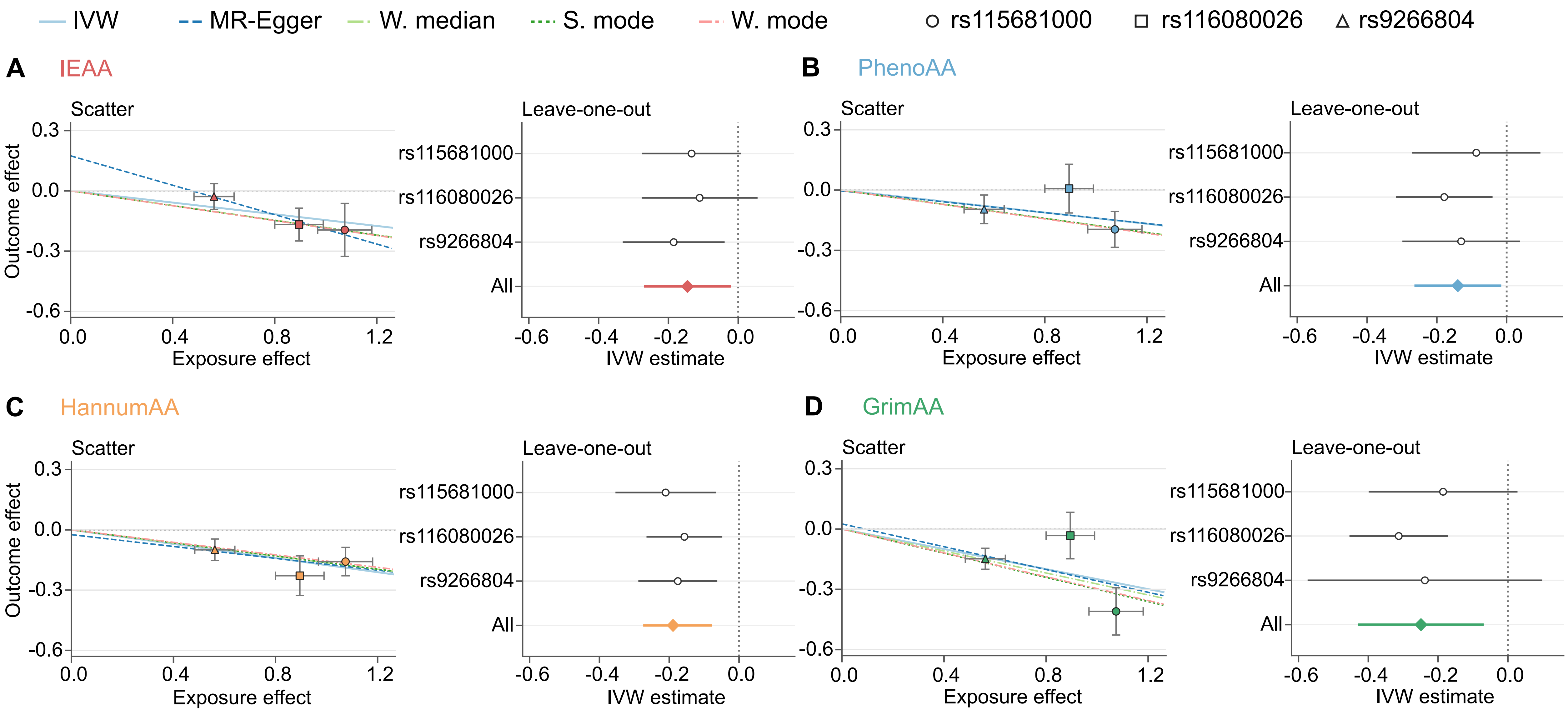
IVW
MR-Egger
W. median
S. mode
W. mode
rs115681000
rs116080026
rs9266804
A
IEAA
B
PhenoAA
C
HannumAA
D
GrimAA
Scatter
Leave-one-out
Outcome effect
Exposure effect
IVW estimate
All
