# Supplementary material for: Shared Genetic Architecture Between Epigenetic Aging and Musculoskeletal Diseases
Source: Genes (Basel). 2026 Jul 28;17(8):878. doi: 10.3390/genes17080878 (PMC13512585; doi:10.3390/genes17080878)
Supplement: Supplementary file 1 [file genes-17-00878-s001.zip › supplementary_figure_S3_revised1.pdf]

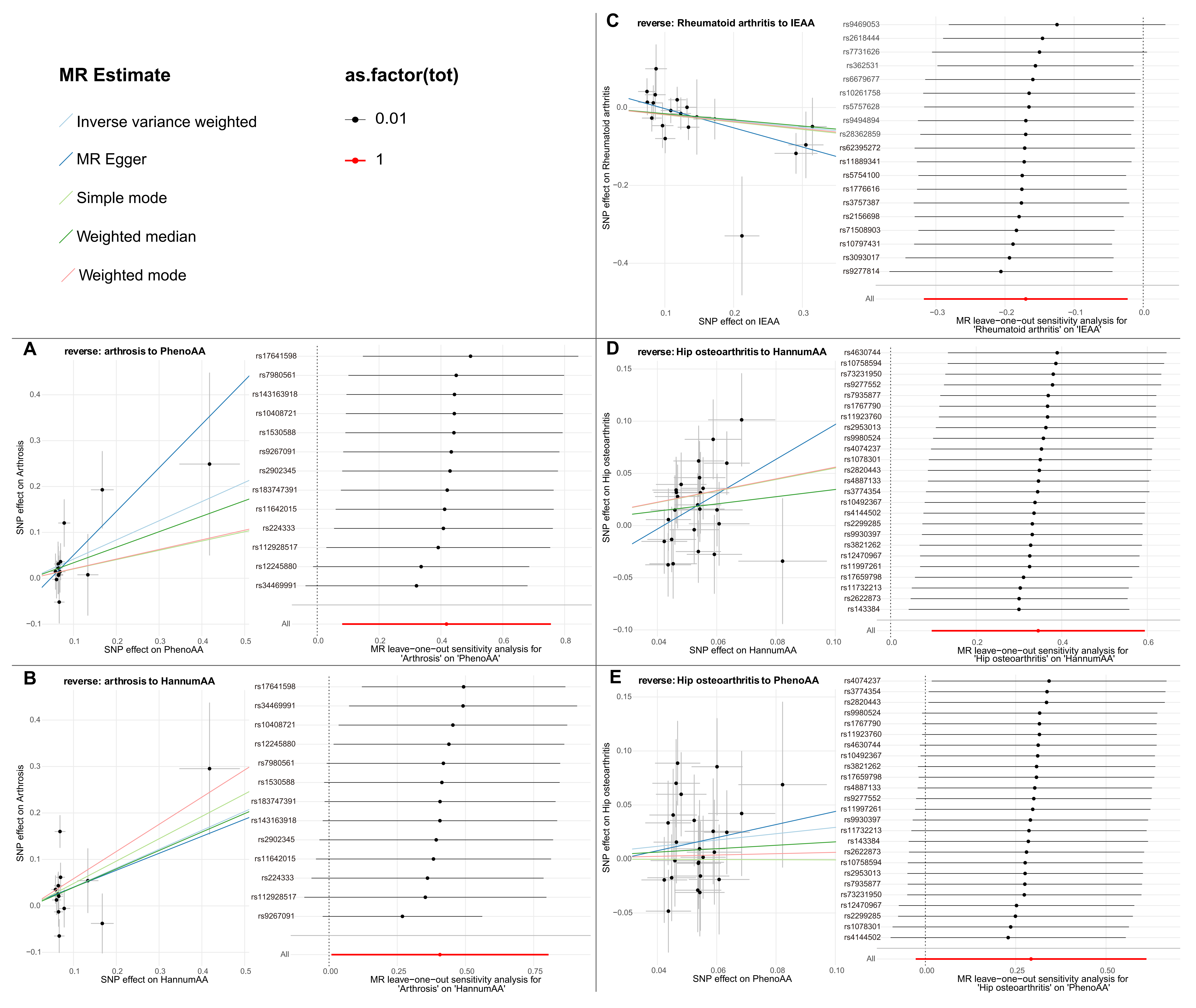
MR Estimate
Inverse variance weighted
MR Egger
Simple mode
Weighted median
Weighted mode
as.factor(tot)
0.01
1
C
reverse: Rheumatoid arthritis to IEAA
SNP effect on Rheumatoid arthritis
SNP effect on IEAA
rs9469053
rs2618444
rs7731626
rs362531
rs6679677
rs10261758
rs5757628
rs9494894
rs28362859
rs62395272
rs11889341
rs5754100
rs1776616
rs3757387
rs2156698
rs71508903
rs10797431
rs3093017
rs9277814
All
MR leave−one−out sensitivity analysis for 'Rheumatoid arthritis' on 'IEAA'
A
reverse: arthrosis to PhenoAA
SNP effect on Arthrosis
SNP effect on PhenoAA
rs17641598
rs7980561
rs143163918
rs10408721
rs1530588
rs9267091
rs2902345
rs183747391
rs11642015
rs224333
rs112928517
rs12245880
rs34469991
All
MR leave−one−out sensitivity analysis for 'Arthrosis' on 'PhenoAA'
D
reverse: Hip osteoarthritis to HannumAA
SNP effect on Hip osteoarthritis
SNP effect on HannumAA
rs4630744
rs10758594
rs73231950
rs9277552
rs7935877
rs1767790
rs11923760
rs2953013
rs9980524
rs4074237
rs1078301
rs2820443
rs4887133
rs3774354
rs10492367
rs4144502
rs2299285
rs9930397
rs3821262
rs12470967
rs11997261
rs17659798
rs11732213
rs2622873
rs143384
All
MR leave−one−out sensitivity analysis for 'Hip osteoarthritis' on 'HannumAA'
B
reverse: arthrosis to HannumAA
SNP effect on Arthrosis
SNP effect on HannumAA
rs17641598
rs34469991
rs10408721
rs12245880
rs7980561
rs1530588
rs183747391
rs143163918
rs2902345
rs11642015
rs224333
rs112928517
rs9267091
All
MR leave−one−out sensitivity analysis for 'Arthrosis' on 'HannumAA'
E
reverse: Hip osteoarthritis to PhenoAA
SNP effect on Hip osteoarthritis
SNP effect on PhenoAA
rs4074237
rs3774354
rs2820443
rs9980524
rs1767790
rs11923760
rs4630744
rs10492367
rs3821262
rs17659798
rs4887133
rs9277552
rs11997261
rs9930397
rs11732213
rs143384
rs2622873
rs10758594
rs2953013
rs7935877
rs73231950
rs12470967
rs2299285
rs1078301
rs4144502
All
MR leave−one−out sensitivity analysis for 'Hip osteoarthritis' on 'PhenoAA'
