# Supplementary material for: Shared Genetic Architecture Between Epigenetic Aging and Musculoskeletal Diseases
Source: Genes (Basel). 2026 Jul 28;17(8):878. doi: 10.3390/genes17080878 (PMC13512585; doi:10.3390/genes17080878)
Supplement: Supplementary file 1 [file genes-17-00878-s001.zip › Supplementary_Figure_S4.pdf]

## Panel A | GrimAA (n = 21)

| SNP | Gene | VEP consequence | Threshold | Per-SNP F | Blood | Immune | Bone/joint | Cancer/clonal | Telomere | Protein | Aging | Steiger ret./eval. | Set C ret./rebuilt | Set C exclude |
|---|---|---|---|---|---|---|---|---|---|---|---|---|---|---|
| rs1008084 | CD164 | intron | GWS | 56.5 | ■ | ■ | | ■ | ■ | ■ | ■ | 10/10 | 0/10 | ✖ |
| rs112937895 | CDH18 | intergenic | Fallback | 51.4 | | | | | | | | 10/10 | 10/10 | ○ |
| rs114450977 | RRP15 | intergenic | Fallback | 35.6 | | | | | | | | 10/10 | 10/10 | ○ |
| rs1499044 | GRM5 | intron | Fallback | 22.6 | | | | | | | | 10/10 | 10/10 | ○ |
| rs17094148 | NKX2-3 | regulatory | Fallback | 23.5 | ■ | ■ | ■ | | ■ | □ | ■ | 9/10 | 0/10 | ✖ |
| rs1828372 | DDR2 | intron | Fallback | 13.0 | ■ | | | | | | | 10/10 | 0/10 | ✖ |
| rs1910560 | CDH2 | intergenic | Fallback | 42.0 | | | | | | | | 10/10 | 10/10 | ○ |
| rs2209425 | SH3GL2 | intron | Fallback | 14.7 | | | | | | | ■ | 9/10 | 9/10 | ○ |
| rs2473606 | AKAP12 | intron | Fallback | 11.0 | | | | | | | | 10/10 | 10/10 | ○ |
| rs2697766 | ALPPL2 | upstream | Fallback | 14.1 | | | | | | | | 9/10 | 9/10 | ○ |
| rs2858830 | FAM26F | upstream | GWS | 19.3 | | | | | | | ■ | 9/9 | 9/10 | ○ |
| rs3931960 | GSDMA | upstream | Fallback | 23.4 | ■ | | | | | | | 0/0 | 0/10 | ✖ |
| rs403269 | GABRG3 | intron | Fallback | 21.9 | | | | | | | | 10/10 | 10/10 | ○ |
| rs613131 | VPS39 | intron | GWS | 17.9 | | | | | | □ | | 10/10 | 10/10 | ○ |
| rs6478340 | TLR4 | intergenic | Fallback | 14.0 | | | | | | | | 10/10 | 10/10 | ○ |
| rs6806687 | RPN1 | intron | Fallback | 36.7 | ■ | ■ | | | ■ | □ | | 10/10 | 0/10 | ✖ |
| rs73950873 | TSN | intergenic | Fallback | 17.2 | | | | | | | | 10/10 | 10/10 | ○ |
| rs78784579 | C3orf35 | intron | GWS | 22.9 | | | | | | | ■ | 9/10 | 9/10 | ○ |
| rs8065978 | PMP22 | intergenic | Fallback | 16.6 | | | | | | | | 10/10 | 10/10 | ○ |
| rs887466 | POU5F1 | intron | Fallback | 21.2 | | | | | | □ | ■ | 7/9 | 7/10 | ○ |
| rs9853301 | PYDC2 | intergenic | Fallback | 20.9 | | | | | | | ■ | 10/10 | 10/10 | ○ |

F = 10

## Panel B | HannumAA (n = 9)

| SNP | Gene | VEP consequence | Threshold | Per-SNP F | Blood | Immune | Smoking | Metabolic | Cardio/lipid | Bone/joint | Cancer/clonal | Protein | Aging | Steiger ret./eval. | Set C ret./rebuilt | Set C exclude |
|---|---|---|---|---|---|---|---|---|---|---|---|---|---|---|---|---|
| rs1005277 | ZNF25 | intergenic | GWS | 83.0 | | | | | | | | | ■ | 10/10 | 10/10 | ○ |
| rs10786282 | TLL2 | upstream | GWS | 98.9 | | | | | | | | | | 10/10 | 10/10 | ○ |
| rs12417758 | CD248 | upstream | GWS | 47.4 | | ■ | | ■ | | ■ | | ■ | ■ | 10/10 | 0/10 | ✖ |
| rs1598856 | NFKB1 | intron | GWS | 39.4 | | ■ | | | ■ | | | ■ | ■ | 10/10 | 0/10 | ✖ |
| rs290805 | KLF14 | upstream | GWS | 32.0 | | | | ■ | | ■ | | | ■ | 10/10 | 0/10 | ✖ |
| rs3093956 | MICB | intron | GWS | 37.6 | ■ | ■ | ■ | ■ | | | ■ | ■ | ■ | 9/10 | 0/10 | ✖ |
| rs34970912 | ZFHX3 | intron | GWS | 30.8 | | | | | | | | | ■ | 10/10 | 10/10 | ○ |
| rs4383328 | FAM49A | intergenic | GWS | 33.0 | ■ | | | | | ■ | | | ■ | 10/10 | 0/10 | ✖ |
| rs4838595 | ARHGAP22 | intron | GWS | 32.3 | | | | | | | | | ■ | 10/10 | 10/10 | ○ |

F = 10

## Panel C | IEAA (n = 20)

| SNP | Gene | VEP consequence | Threshold | Per-SNP F | Blood | Immune | Metabolic | Glycemic | Cardio/lipid | Bone/joint | Cancer/clonal | Telomere | Protein | Aging | Steiger ret./eval. | Set C ret./rebuilt | Set C exclude |
|---|---|---|---|---|---|---|---|---|---|---|---|---|---|---|---|---|---|
| rs10778518 | C12orf23 | downstream | GWS | 45.0 | | | ■ | | | | | | | ■ | 10/10 | 0/10 | ✖ |
| rs1126828 | RPN1 | synonymous | GWS | 49.2 | ■ | | | | | | ■ | | | ■ | 10/10 | 0/10 | ✖ |
| rs11911381 | RIPPLY3 | intergenic | GWS | 46.9 | | | | | | | | | | | 10/10 | 10/10 | ○ |
| rs12043492 | AKIRIN1 | 5' UTR | GWS | 30.9 | | | | | | | | | | ■ | 10/10 | 10/10 | ○ |
| rs12145290 | CD34 | intron | GWS | 26.7 | ■ | ■ | | | | ■ | | | ■ | ■ | 10/10 | 0/10 | ✖ |
| rs12666349 | PPP1R17 | intron | GWS | 51.7 | | | | | | | | | | ■ | 10/10 | 10/10 | ○ |
| rs12903325 | ATP8B4 | intron | GWS | 18.5 | ■ | | | | | | | ■ | □ | ■ | 10/10 | 0/10 | ✖ |
| rs1849209 | SETBP1 | intergenic | GWS | 42.0 | ■ | | | | | ■ | ■ | ■ | □ | ■ | 10/10 | 0/10 | ✖ |
| rs1932529 | RERE | intron | GWS | 41.6 | | | | | | | | | | | 3/3 | 3/10 | ○ |
| rs2517717 | HLA-A | downstream | GWS | 63.8 | ■ | ■ | | | | | | | ■ | | 8/10 | 0/10 | ✖ |
| rs2853677 | TERT | intron | GWS | 41.5 | ■ | | | | | | ■ | ■ | □ | ■ | 10/10 | 0/10 | ✖ |
| rs4680588 | TRIM59 | intron | GWS | 61.5 | ■ | | ■ | ■ | ■ | ■ | ■ | | | ■ | 10/10 | 0/10 | ✖ |
| rs4832591 | FAM49A | intron | GWS | 42.9 | | | | | | | | | | | 10/10 | 10/10 | ○ |
| rs589534 | EDARADD | intron | GWS | 22.0 | | | | | | | | | | ■ | 10/10 | 10/10 | ○ |
| rs59689077 | DIRAS3 | intron | GWS | 30.0 | | | | | | | | | | ■ | 10/10 | 10/10 | ○ |
| rs61888888 | P2RX3 | upstream | GWS | 77.6 | | | | | ■ | | | | | ■ | 10/10 | 0/10 | ✖ |
| rs75243280 | IL17RA | synonymous | GWS | 60.3 | | | | | | | | | | ■ | 3/3 | 3/10 | ○ |
| rs76244256 | TPMT | intron | GWS | 138.2 | | | ■ | | | | | | □ | ■ | 10/10 | 0/10 | ✖ |
| rs764199 | SELP | intron | GWS | 56.8 | | | | | | | | | | ■ | 0/0 | 0/10 | ○ |
| rs9846208 | TSC22D2 | intron | GWS | 23.1 | | | | | | | | | | | 10/10 | 10/10 | ○ |

F = 10

## Panel D | PhenoAA (n = 11)

| SNP | Gene | VEP consequence | Threshold | Per-SNP F | Blood | Metabolic | Cardio/lipid | Cancer/clonal | Telomere | Protein | Aging | Steiger ret./eval. | Set C ret./rebuilt | Set C exclude |
|---|---|---|---|---|---|---|---|---|---|---|---|---|---|---|
| rs11190127 | NKX2-3 | intergenic | GWS | 39.1 | ■ | | | | | | ■ | 3/3 | 0/10 | ✖ |
| rs11253338 | DIP2C | intergenic | GWS | 33.2 | | | | | | | ■ | 10/10 | 10/10 | ○ |
| rs116853700 | MSI2 | intron | GWS | 32.0 | ■ | | | ■ | | ■ | ■ | 10/10 | 0/10 | ✖ |
| rs1990053 | PURB | nc transcript exon | GWS | 44.9 | ■ | ■ | ■ | ■ | | ■ | ■ | 10/10 | 0/10 | ✖ |
| rs3829957 | ASPA | 5' UTR | GWS | 62.0 | | | | | | | ■ | 10/10 | 10/10 | ○ |
| rs6531114 | FAM49A | downstream | GWS | 35.9 | ■ | | | ■ | ■ | | | 10/10 | 0/10 | ✖ |
| rs678553 | EDARADD | intron | GWS | 62.8 | | | | | | | ■ | 10/10 | 10/10 | ○ |
| rs7228835 | SETBP1 | intron | GWS | 71.1 | ■ | | | ■ | ■ | □ | ■ | 10/10 | 0/10 | ✖ |
| rs73028070 | UBASH3B | 3' UTR | GWS | 31.8 | | | | | | | ■ | 9/10 | 9/10 | ○ |
| rs75194173 | KDM1B | intron | GWS | 90.3 | | | | | | | | 10/10 | 10/10 | ○ |
| rs752223 | CYP2J2 | regulatory | GWS | 60.5 | | | | | | | ■ | 10/10 | 10/10 | ○ |

F = 10

■ High-confidence domain evidence  □ Moderate-confidence domain evidence  —×— Low-confidence domain evidence  ✖ Excluded from corrected Set C
