# Supplementary material for: Shared Genetic Architecture Between Epigenetic Aging and Musculoskeletal Diseases
Source: Genes (Basel). 2026 Jul 28;17(8):878. doi: 10.3390/genes17080878 (PMC13512585; doi:10.3390/genes17080878)
Supplement: Supplementary file 1 [file genes-17-00878-s001.zip › Supplementary_Figure_S5.pdf]

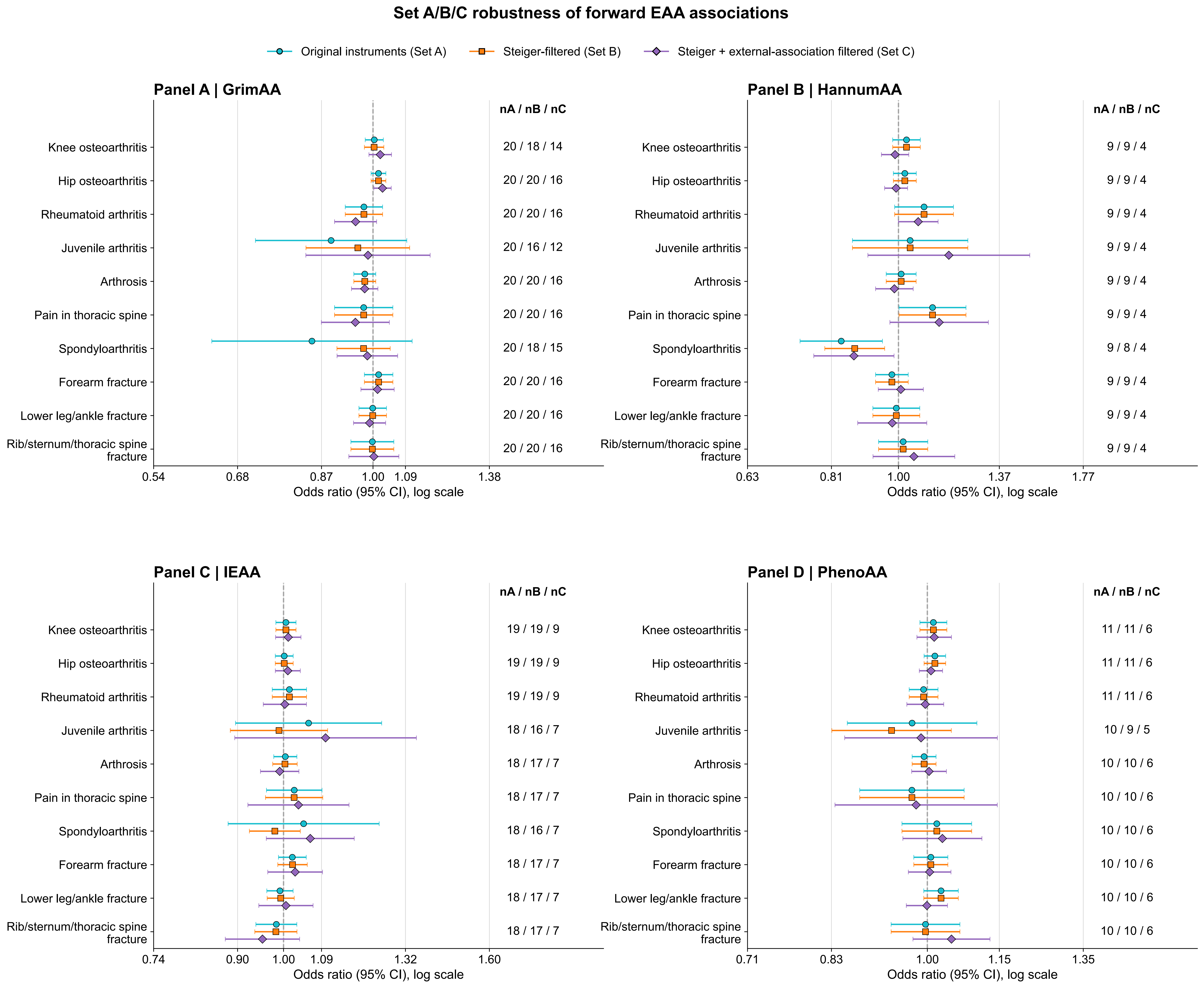

Set A/B/C robustness of forward EAA associations
Original instruments (Set A)
Steiger-filtered (Set B)
Steiger + external-association filtered (Set C)
Panel A | GrimAA
nA / nB / nC
Knee osteoarthritis
20 / 18 / 14
Hip osteoarthritis
20 / 20 / 16
Rheumatoid arthritis
20 / 20 / 16
Juvenile arthritis
20 / 16 / 12
Arthrosis
20 / 20 / 16
Pain in thoracic spine
20 / 20 / 16
Spondyloarthritis
20 / 18 / 15
Forearm fracture
20 / 20 / 16
Lower leg/ankle fracture
20 / 20 / 16
Rib/sternum/thoracic spine fracture
20 / 20 / 16
0.54
0.68
0.87
1.00
1.09
1.38
Odds ratio (95% CI), log scale
Panel B | HannumAA
nA / nB / nC
Knee osteoarthritis
9 / 9 / 4
Hip osteoarthritis
9 / 9 / 4
Rheumatoid arthritis
9 / 9 / 4
Juvenile arthritis
9 / 9 / 4
Arthrosis
9 / 9 / 4
Pain in thoracic spine
9 / 9 / 4
Spondyloarthritis
9 / 8 / 4
Forearm fracture
9 / 9 / 4
Lower leg/ankle fracture
9 / 9 / 4
Rib/sternum/thoracic spine fracture
9 / 9 / 4
0.63
0.81
1.00
1.37
1.77
Odds ratio (95% CI), log scale
Panel C | IEAA
nA / nB / nC
Knee osteoarthritis
19 / 19 / 9
Hip osteoarthritis
19 / 19 / 9
Rheumatoid arthritis
19 / 19 / 9
Juvenile arthritis
18 / 16 / 7
Arthrosis
18 / 17 / 7
Pain in thoracic spine
18 / 17 / 7
Spondyloarthritis
18 / 16 / 7
Forearm fracture
18 / 17 / 7
Lower leg/ankle fracture
18 / 17 / 7
Rib/sternum/thoracic spine fracture
18 / 17 / 7
0.74
0.90
1.00
1.09
1.32
1.60
Odds ratio (95% CI), log scale
Panel D | PhenoAA
nA / nB / nC
Knee osteoarthritis
11 / 11 / 6
Hip osteoarthritis
11 / 11 / 6
Rheumatoid arthritis
11 / 11 / 6
Juvenile arthritis
10 / 9 / 5
Arthrosis
10 / 10 / 6
Pain in thoracic spine
10 / 10 / 6
Spondyloarthritis
10 / 10 / 6
Forearm fracture
10 / 10 / 6
Lower leg/ankle fracture
10 / 10 / 6
Rib/sternum/thoracic spine fracture
10 / 10 / 6
0.71
0.83
1.00
1.15
1.35
Odds ratio (95% CI), log scale
