# Supplementary material for: Shared Genetic Architecture Between Epigenetic Aging and Musculoskeletal Diseases
Source: Genes (Basel). 2026 Jul 28;17(8):878. doi: 10.3390/genes17080878 (PMC13512585; doi:10.3390/genes17080878)
Supplement: Supplementary file 1 [file genes-17-00878-s001.zip › Supplementary_Figure_S6.pdf]

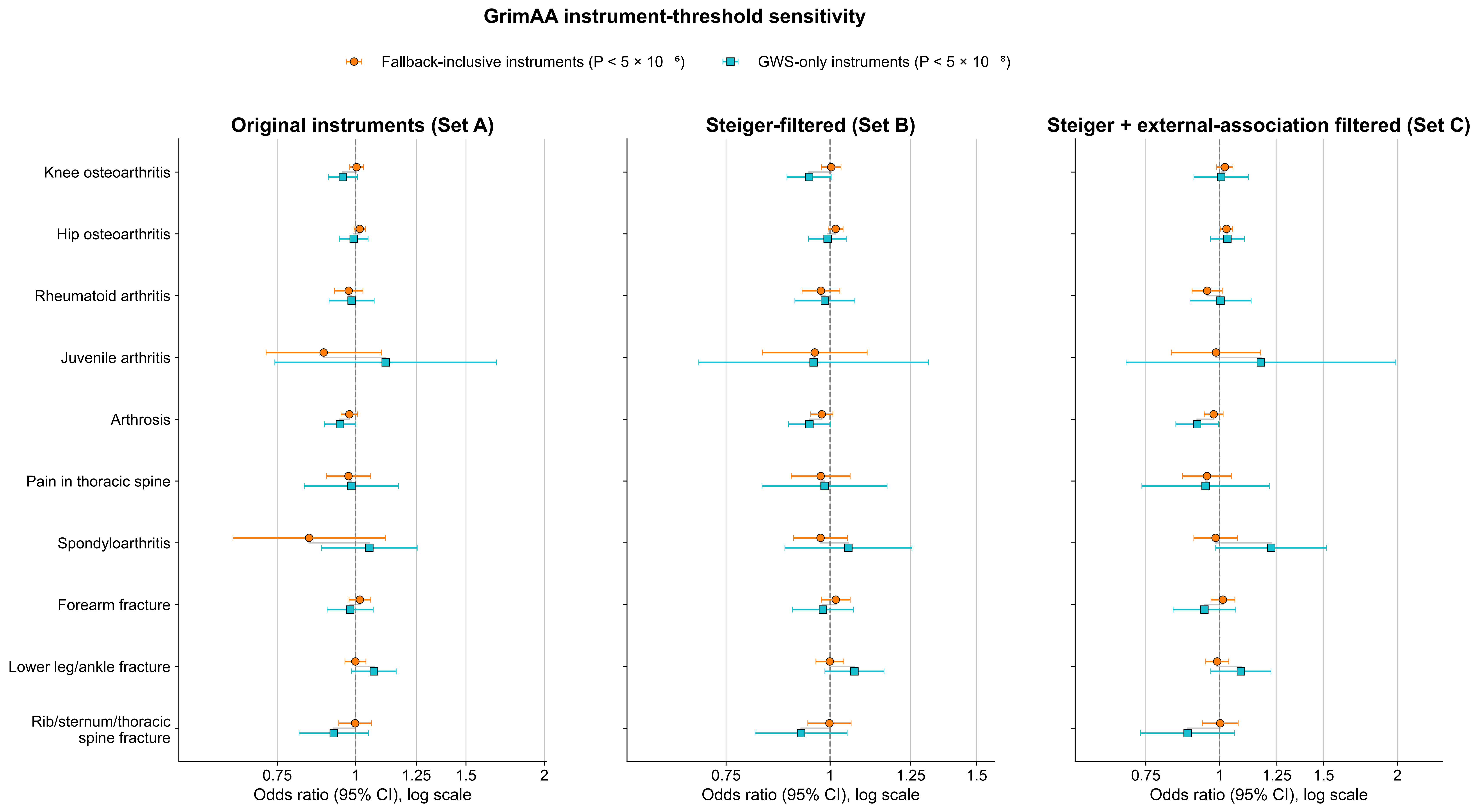

GrimAA instrument-threshold sensitivity
Fallback-inclusive instruments (P < 5 × 10⁻⁶)
GWS-only instruments (P < 5 × 10⁻⁸)
Original instruments (Set A)
Steiger-filtered (Set B)
Steiger + external-association filtered (Set C)
Knee osteoarthritis
Hip osteoarthritis
Rheumatoid arthritis
Juvenile arthritis
Arthrosis
Pain in thoracic spine
Spondyloarthritis
Forearm fracture
Lower leg/ankle fracture
Rib/sternum/thoracic spine fracture
0.75
1
1.25
1.5
2
Odds ratio (95% CI), log scale
