# Supplementary material for: Shared Genetic Architecture Between Epigenetic Aging and Musculoskeletal Diseases
Source: Genes (Basel). 2026 Jul 28;17(8):878. doi: 10.3390/genes17080878 (PMC13512585; doi:10.3390/genes17080878)
Supplement: Supplementary file 1 [file genes-17-00878-s001.zip › Supplementary_Figure_S7.pdf]

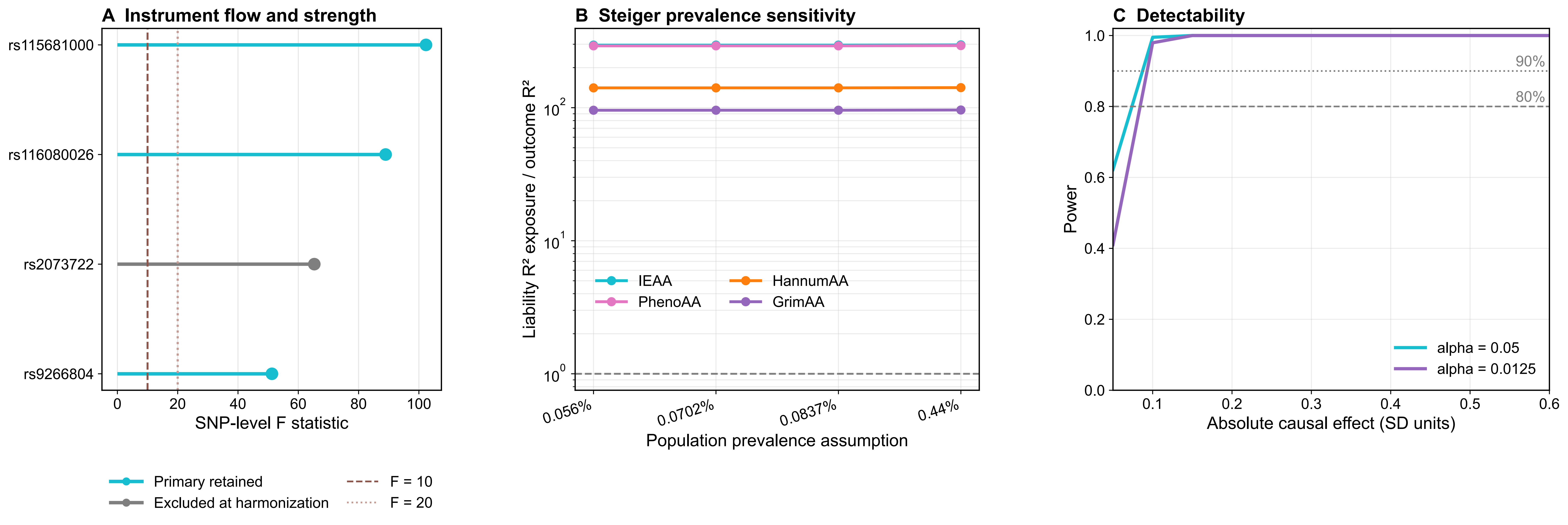

A Instrument flow and strength
rs115681000
rs116080026
rs2073722
rs9266804
0
20
40
60
80
100
SNP-level F statistic
Primary retained
Excluded at harmonization
F = 10
F = 20
B Steiger prevalence sensitivity
Liability R² exposure / outcome R²
10^2
10^1
10^0
IEAA
PhenoAA
HannumAA
GrimAA
0.056%
0.0702%
0.0837%
0.44%
Population prevalence assumption
C Detectability
Power
1.0
0.8
0.6
0.4
0.2
0.0
90%
80%
alpha = 0.05
alpha = 0.0125
0.1
0.2
0.3
0.4
0.5
0.6
Absolute causal effect (SD units)
