# Supplementary material for: Shared Genetic Architecture Between Epigenetic Aging and Musculoskeletal Diseases
Source: Genes (Basel). 2026 Jul 28;17(8):878. doi: 10.3390/genes17080878 (PMC13512585; doi:10.3390/genes17080878)
Supplement: Supplementary file 1 [file genes-17-00878-s001.zip › supplementary_figure_S8_revised1.pdf]

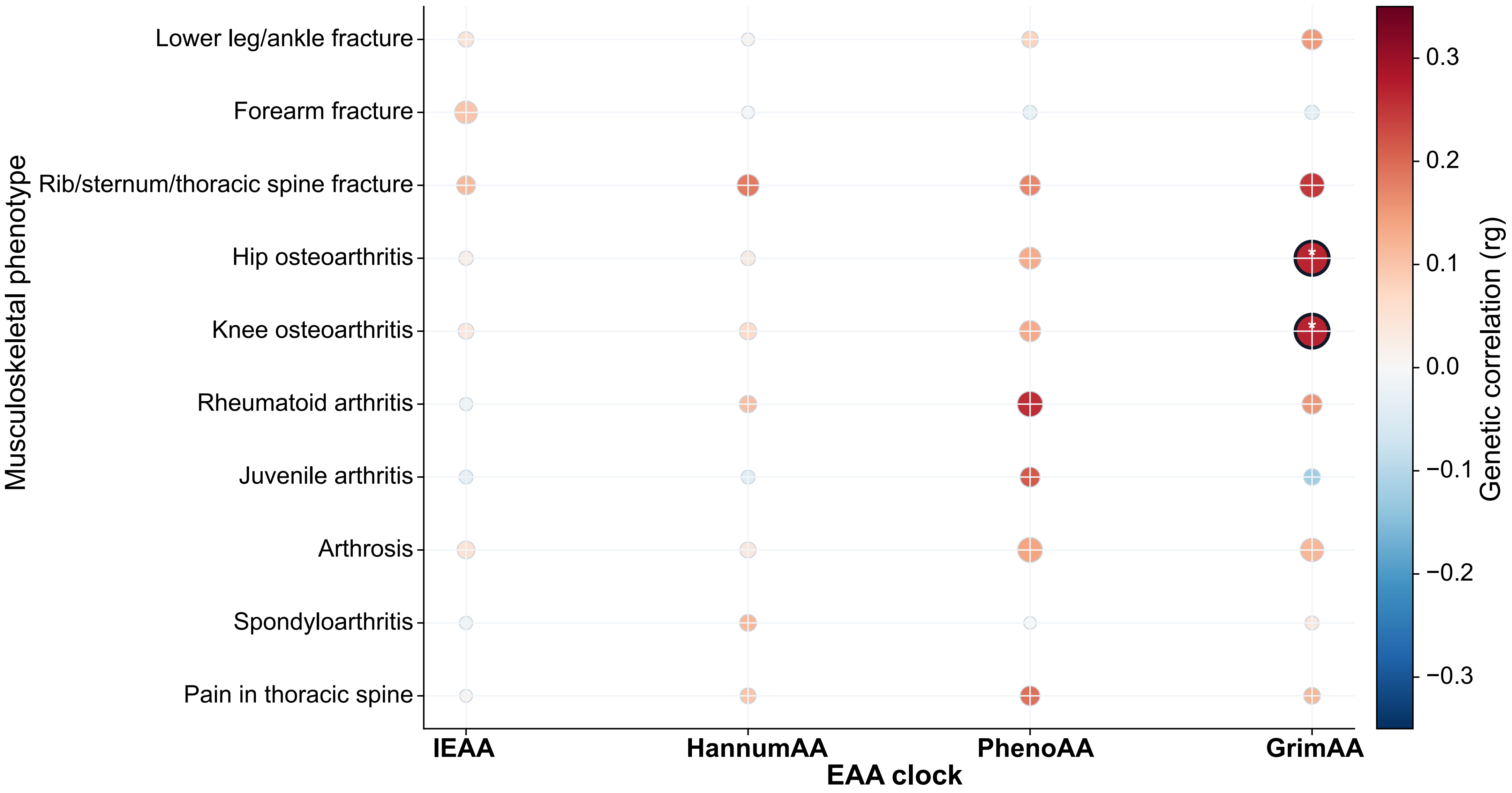
Musculoskeletal phenotype
Lower leg/ankle fracture
Forearm fracture
Rib/sternum/thoracic spine fracture
Hip osteoarthritis
Knee osteoarthritis
Rheumatoid arthritis
Juvenile arthritis
Arthrosis
Spondyloarthritis
Pain in thoracic spine
IEAA
HannumAA
PhenoAA
GrimAA
EAA clock
Genetic correlation (rg)
0.3
0.2
0.1
0.0
−0.1
−0.2
−0.3
*
*
