# Supplementary material for: Shared Genetic Architecture Between Epigenetic Aging and Musculoskeletal Diseases
Source: Genes (Basel). 2026 Jul 28;17(8):878. doi: 10.3390/genes17080878 (PMC13512585; doi:10.3390/genes17080878)
Supplement: Supplementary file 1 [file genes-17-00878-s001.zip › supplementary_figure_S9_revised1.pdf]

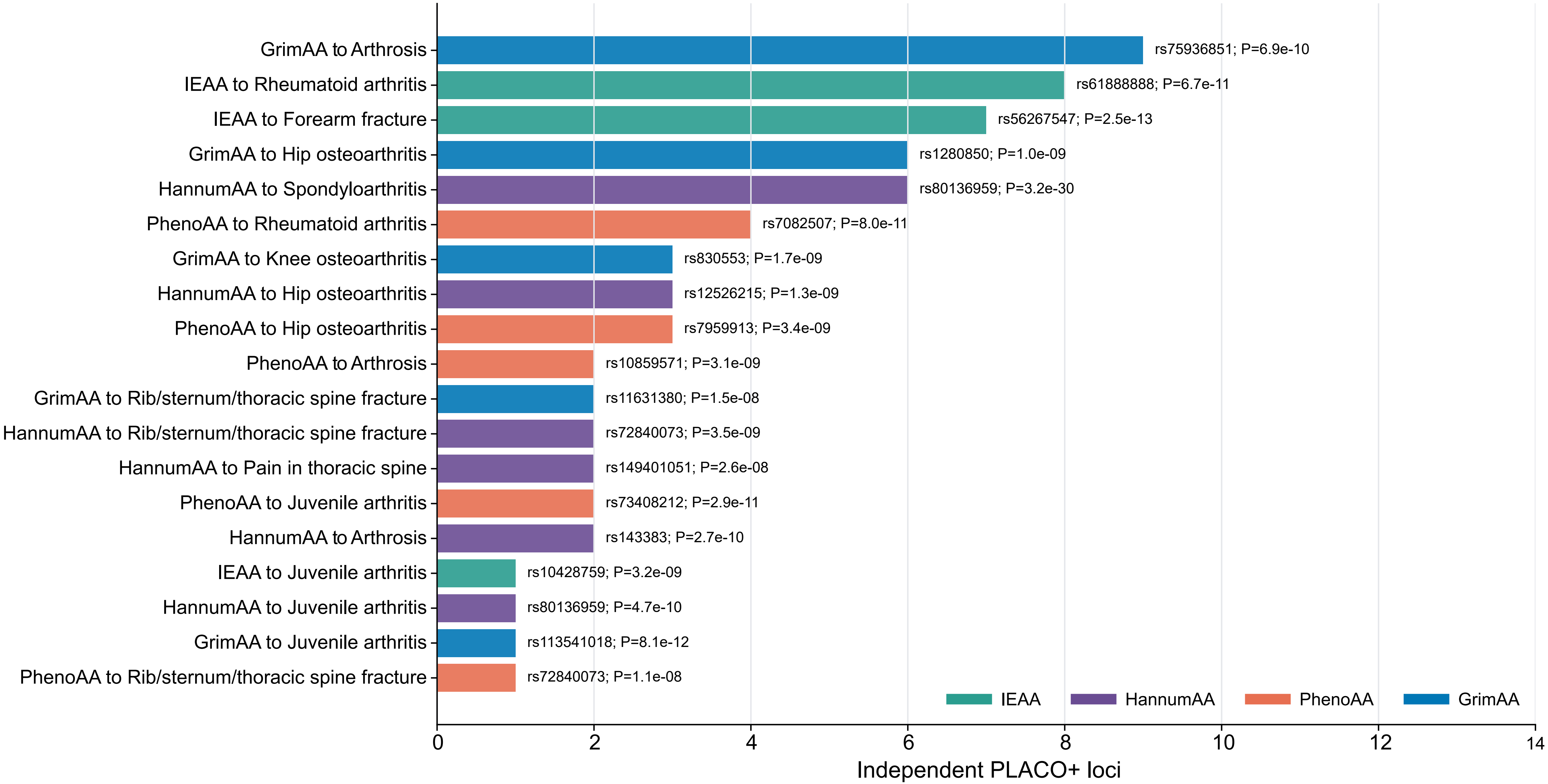

GrimAA to Arthrosis
IEAA to Rheumatoid arthritis
IEAA to Forearm fracture
GrimAA to Hip osteoarthritis
HannumAA to Spondyloarthritis
PhenoAA to Rheumatoid arthritis
GrimAA to Knee osteoarthritis
HannumAA to Hip osteoarthritis
PhenoAA to Hip osteoarthritis
PhenoAA to Arthrosis
GrimAA to Rib/sternum/thoracic spine fracture
HannumAA to Rib/sternum/thoracic spine fracture
HannumAA to Pain in thoracic spine
PhenoAA to Juvenile arthritis
HannumAA to Arthrosis
IEAA to Juvenile arthritis
HannumAA to Juvenile arthritis
GrimAA to Juvenile arthritis
PhenoAA to Rib/sternum/thoracic spine fracture
rs75936851; P=6.9e-10
rs61888888; P=6.7e-11
rs56267547; P=2.5e-13
rs1280850; P=1.0e-09
rs80136959; P=3.2e-30
rs7082507; P=8.0e-11
rs830553; P=1.7e-09
rs12526215; P=1.3e-09
rs7959913; P=3.4e-09
rs10859571; P=3.1e-09
rs11631380; P=1.5e-08
rs72840073; P=3.5e-09
rs149401051; P=2.6e-08
rs73408212; P=2.9e-11
rs143383; P=2.7e-10
rs10428759; P=3.2e-09
rs80136959; P=4.7e-10
rs113541018; P=8.1e-12
rs72840073; P=1.1e-08
IEAA
HannumAA
PhenoAA
GrimAA
0
2
4
6
8
10
12
14
Independent PLACO+ loci
